# Supplementary material for: Universal promoter scanning by Pol II during transcription initiation in Saccharomyces cerevisiae
Source: Genome Biol. 2020 Jun 2;21:132. doi: 10.1186/s13059-020-02040-0 (PMC7265651; doi:10.1186/s13059-020-02040-0)
Supplement: Supplementary file 1 — Additional file 1. Supplemental Fig. S1-S10 and legends. [file 13059_2020_2040_MOESM1_ESM.docx]

**SUPPLEMENTAL FIGURES AND LEGENDS**

**
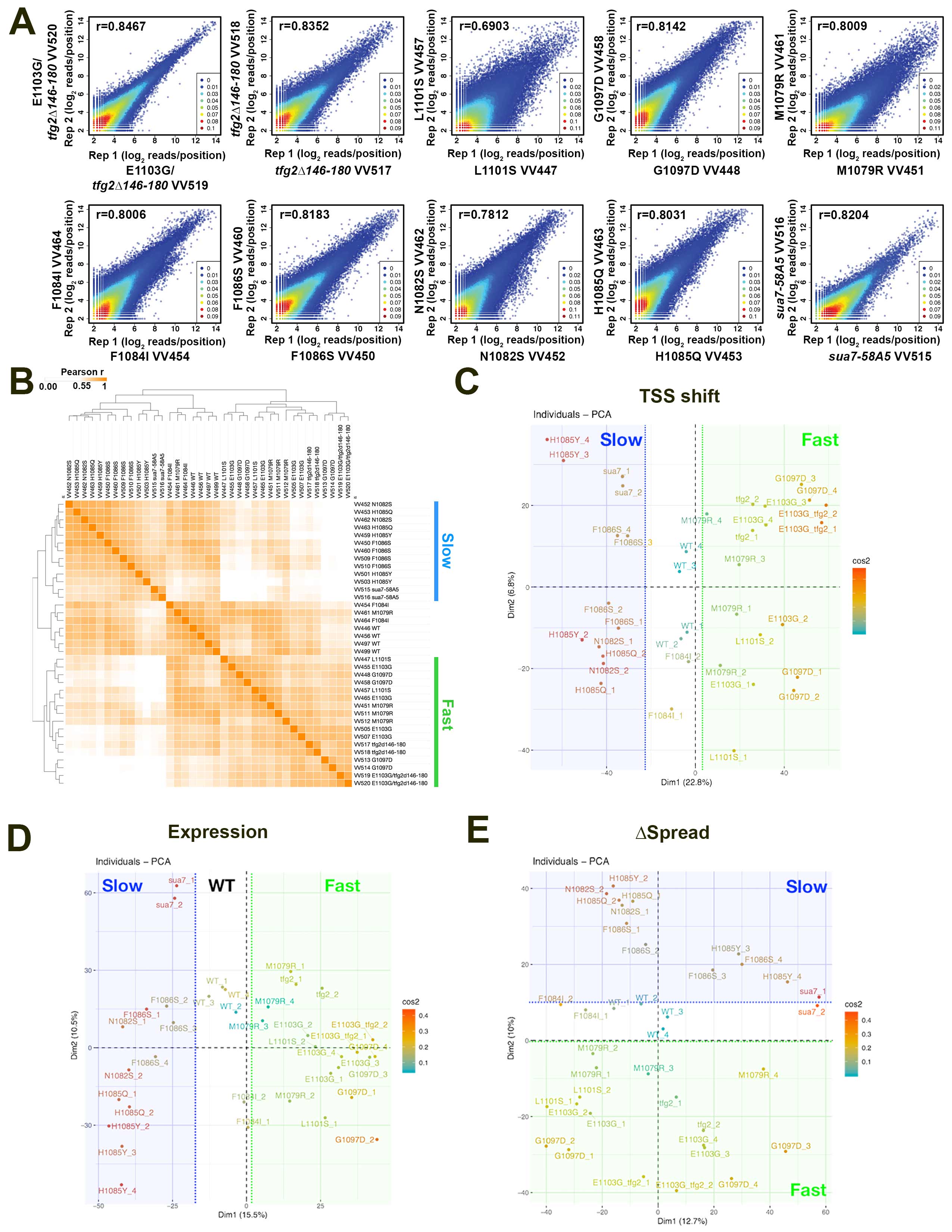
**

**
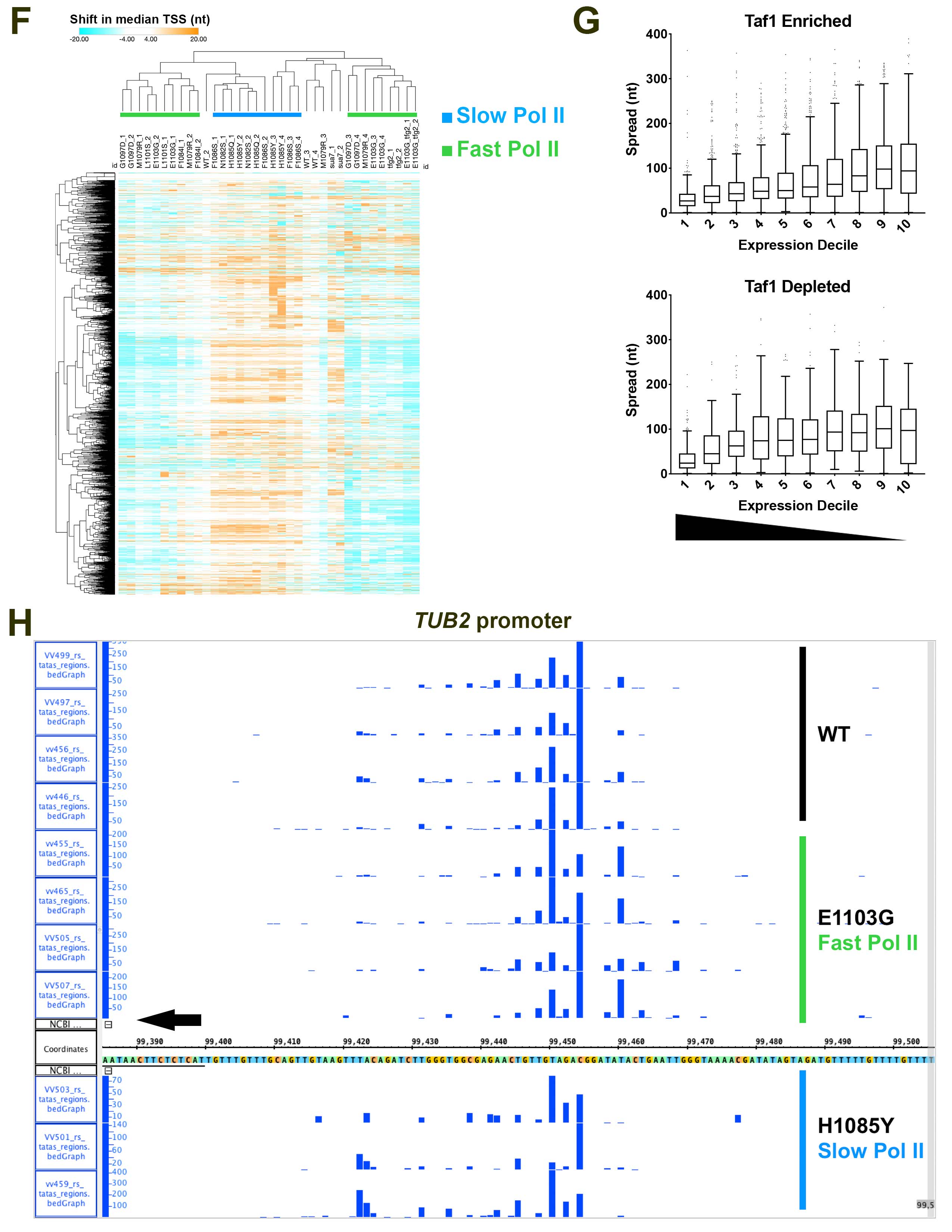
 Fig. S1.** Analysis of TSS-seq replicates. **a.** Example correlation plots for biological replicate TSS-seq libraries. Plots show all genome positions with ≥ 3 reads in each library. Pearson r are listed on each plot. Color scale on the heat scatter plots represent estimated kernel density of the plotted points. **b.** Pearson r correlation coefficients for all TSS-seq library comparisons (examples in A) displayed in a hierarchically clustered heat map. Here, replicates were compared for TSS-seq reads in promoter regions. Promoter regions in this analysis were defined by Rhee and Pugh predicted 8-mer TATA or TATA-like core promoter element position +/- 200 nucleotides upstream and downstream (n=6044). VV numbers represent individual TSS-seq library designations. Libraries VV446-465 represent replicates from one sequencing run (batch one) and VV497-520 represent a separate sequencing run (batch two). Clustering distinguishes two major classes of TSS-seq libraries correlating with upstream TSS-shifting and downstream TSS-shifting mutants. **c.** PCA analysis for individual shifts of TSS medians over all promoters over 200 reads in the aggregated WT data (n=3494) for individual replicate libraries. Dimension one distinguishes between upstream shifting and downstream shifting initiation mutants across all replicates. Dimension two separates the two sequencing batches (batch two libraries are above *y*=0 and batch one libraries are below *y*=0). **d.** PCA analysis for promoter expression for promoters over 200 reads in the aggregated WT data (n=3494) for individual replicate libraries. Dimension one distinguishes between upstream shifting and downstream shifting initiation mutants across all replicates. **e.** PCA analysis for ∆ TSS Spread for promoters over 200 reads in the aggregated WT data (n=3494) for individual replicate libraries. Dimension two distinguishes between upstream shifting and downstream shifting initiation mutants across all replicates. Dimension one appears to distinguish primarily between two sequence batches (batch one on left, batch two on right). **f.** Heat map for TSS shifts for individual promoters over 200 reads in the aggregated WT data (n=3494, *y*-axis) determined independently in biological replicate TSS-seq libraries (n=2-4 per strain, *x*-axis). Slow and fast Pol II mutants are distinguished by large bias for downstream (positive, orange) or upstream (negative, cyan) shifts in median TSS position. **g.** Spread of TSSs as determined by the width of the 10-90^th^ percentiles of the TSSs for individual promoters (Taf1 Enriched (n=2726) or Taf1 Depleted (n=768)) inversely correlate with expression. The highest expressed promoters on average have the most focused promoters. Box plots are Tukey plots (see Methods). **h.** Example TSS-seq reads for WT, *rpb1* H1085Y, and *rpb1* E1103G biological replicate TSS-seq libraries at the *TUB2* promoter (n=4,3,4, respectively). The *TUB2* ATG is on the minus strand and designated by the arrow in the lower left.


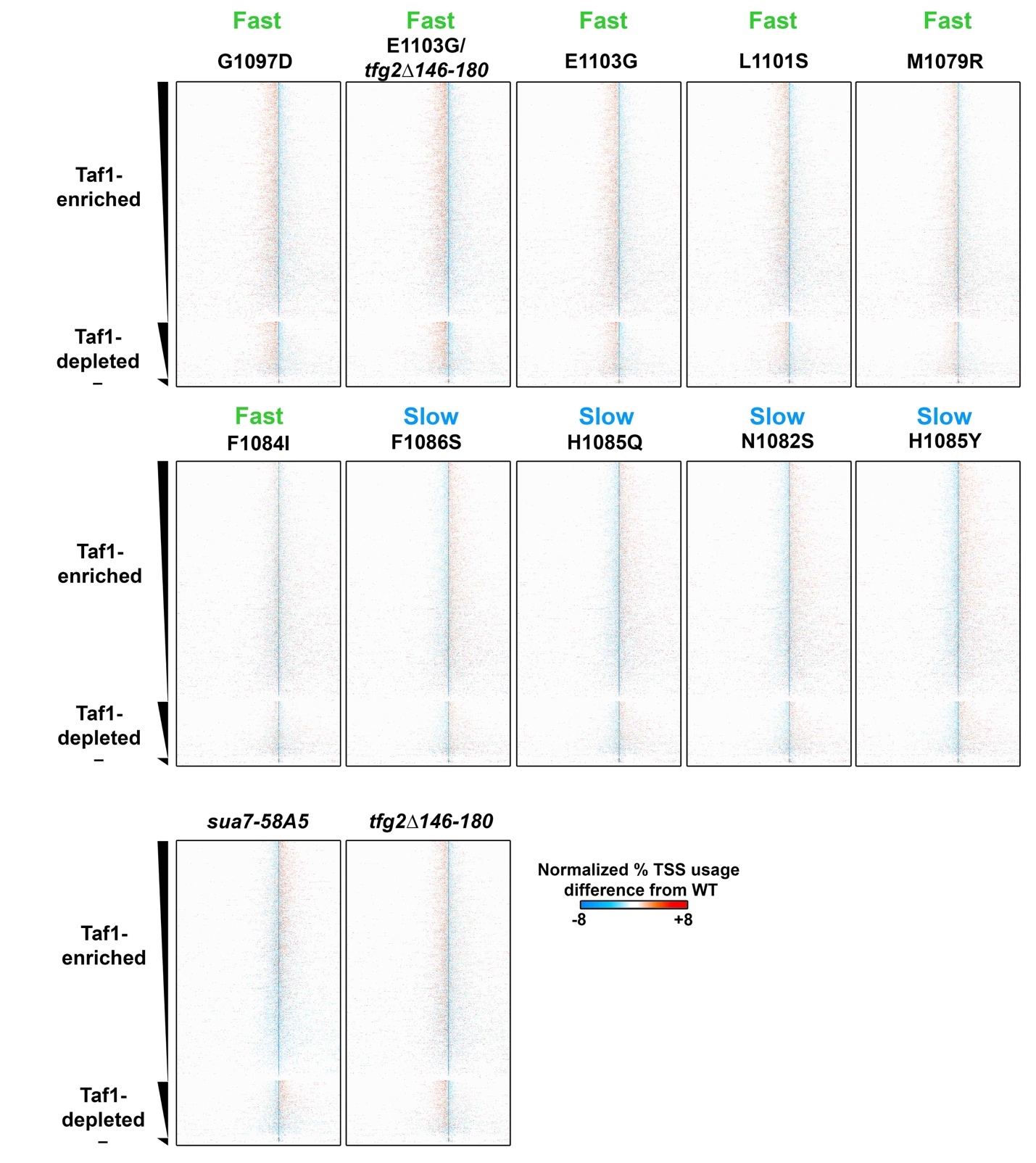


**Fig. S2.** Polar effects on TSS distributions observed for majority of TSS-usage-affecting mutants genome wide. Heat maps as in **Fig. 2a**. *rpb1* H1085Y and *rpb1* E1103G maps from **Fig. 2a** shown here for comparison with all other heat maps. Maps are arranged from strongly upstream shifting to strongly downstream shifting (top left to middle right). Downstream shifting *sua7-58A5* and upstream shifting *tfg2∆146-180* GTF mutants are shown in bottom row.


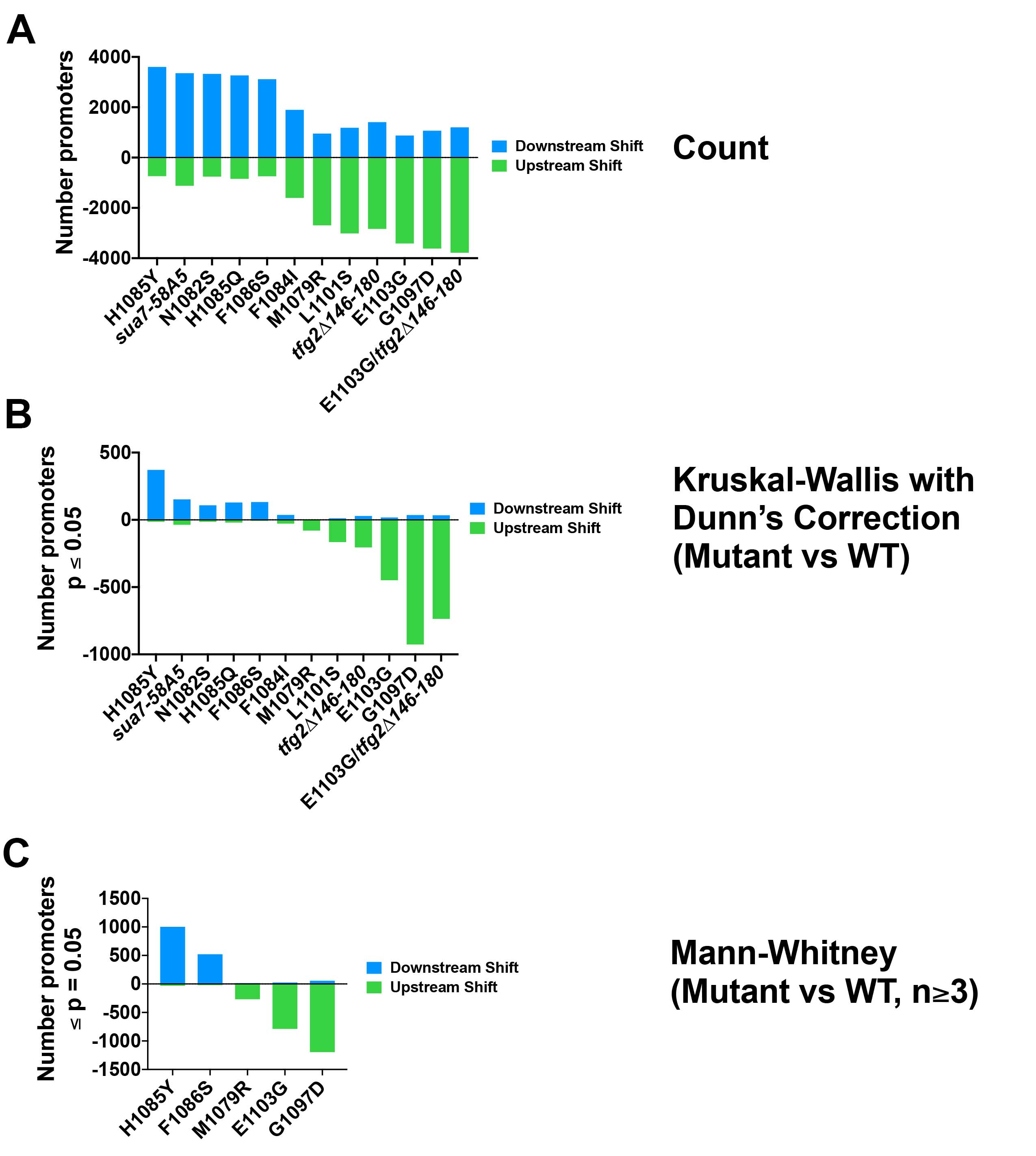


**Fig. S3.** Analysis of promoter level effects of TSS-shifting mutants. **a.** Very large bias in direction of TSS shift depending on mutant class. Counts of promoters with upstream shifts are shown below the *x*-axis (negative numbers) and counts of promoters with downstream shifts are shown above the *x*-axis. **b.** Statistical analysis of significant TSS shifts at individual promoters. TSS shifts for individual promoters across biological replicates for specific mutants were compared to the TSS shifts determined for individual promoters across biological replicates for WT. TSS shifts for each promoter were determined by the median WT TSS position determined from the aggregated WT TSS-seq data. Numbers shown are the individual promoters shifted significantly upstream (negative count) or downstream (positive count) as determined by the Kruskal-Wallis test with Dunn’s correction for multiple comparisons (p≤0.05). The test was performed each promoter, taking into account TSS shift data for all strains (n=2-4**).** **c.** Mann–Whitney *U* test for TSS shifts at individual promoters as in B, but for each strain where replicates n≥3, which were individually compared with WT (n=4 replicates). Numbers shown are the individual promoters shifted significantly upstream (negative count) or downstream (positive count) in TSS-shifting mutant yeast strains.


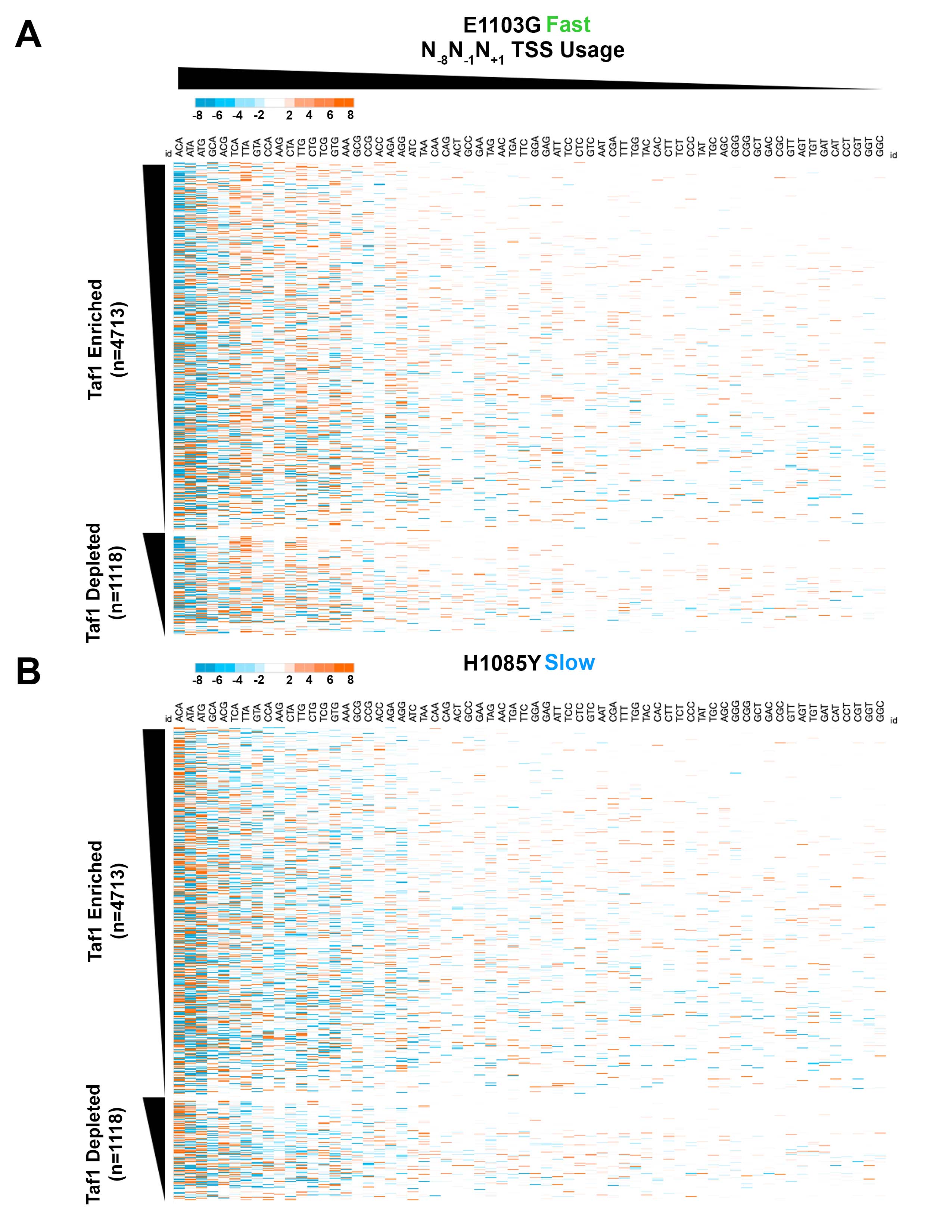

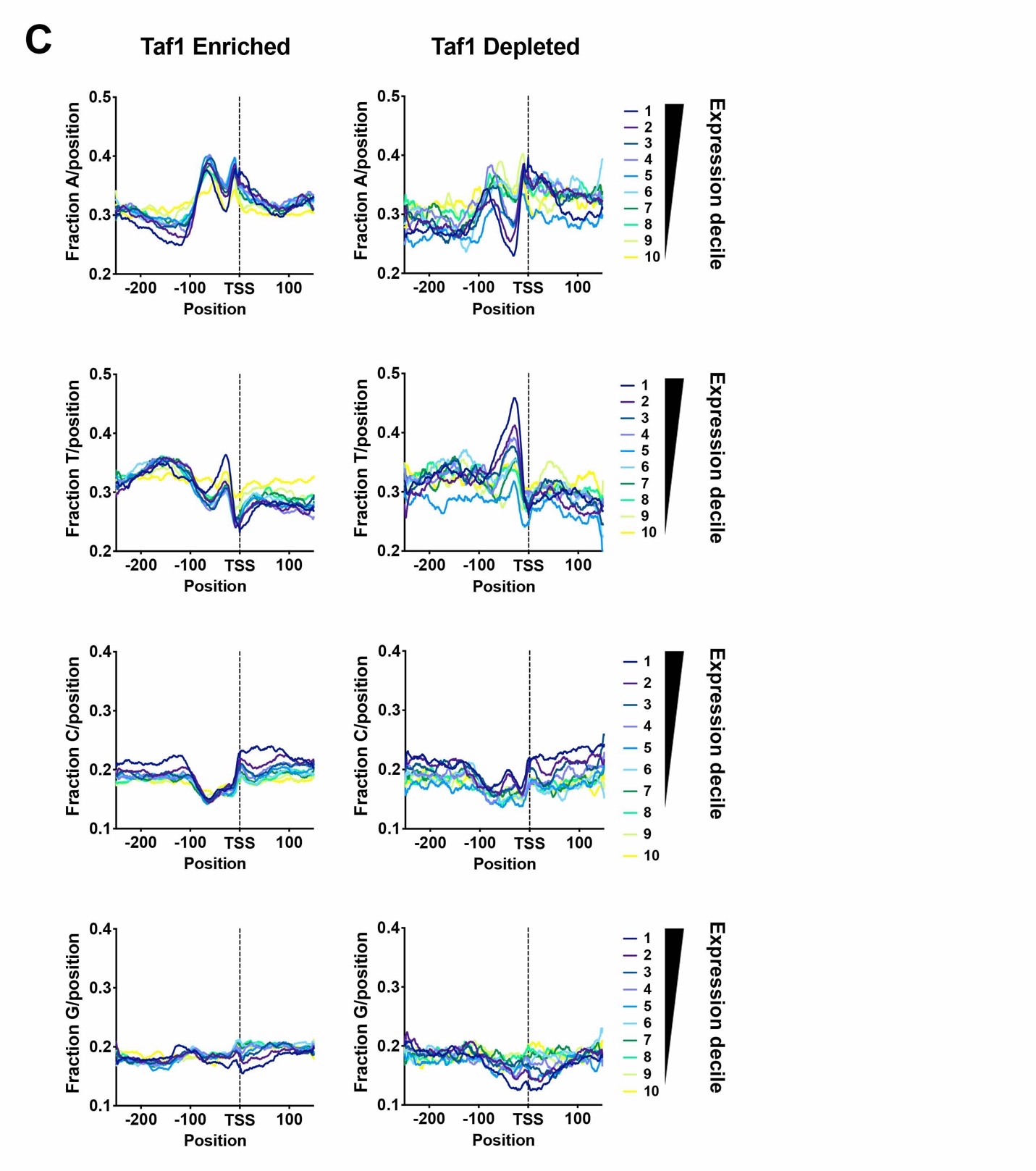


**Fig. S4.** Effects of *rpb1* H1085Y and *rpb1* E1103G mutants on TSS motif usage for N_-8_Y_-1_R_+1_ motifs at the individual promoter level. **a and b.** Heat maps illustrating differences in percent motif usage for individual promoters (*y*-axis) for the 64 N_-8_Y_-1_R_+1_ motifs (*x*-axis) in *rpb1* E1103G (**a**) or *rpb1* H1085Y (**b**) are shown. Motifs are rank ordered based on overall usage across genome in WT yeast (high to low from left to right) and promoters are separated into Taf1 Enriched and Taf1 Depleted classes and rank ordered within class by expression (high to low from top to bottom). **c.** Distribution of bases on the top promoter strand for Taf1 Enriched or Taf1 Depleted promoters, separated by expression decile in WT cells.


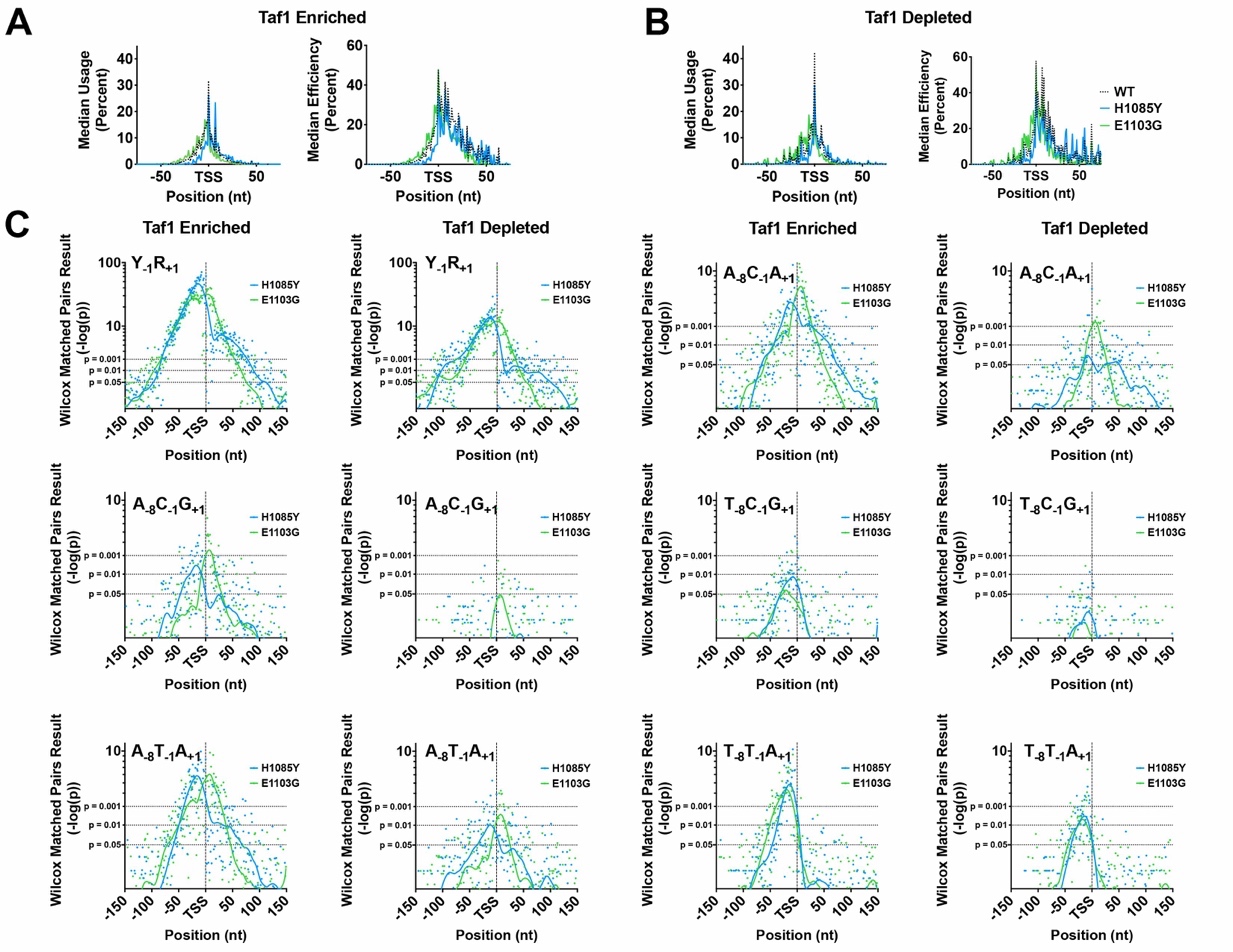

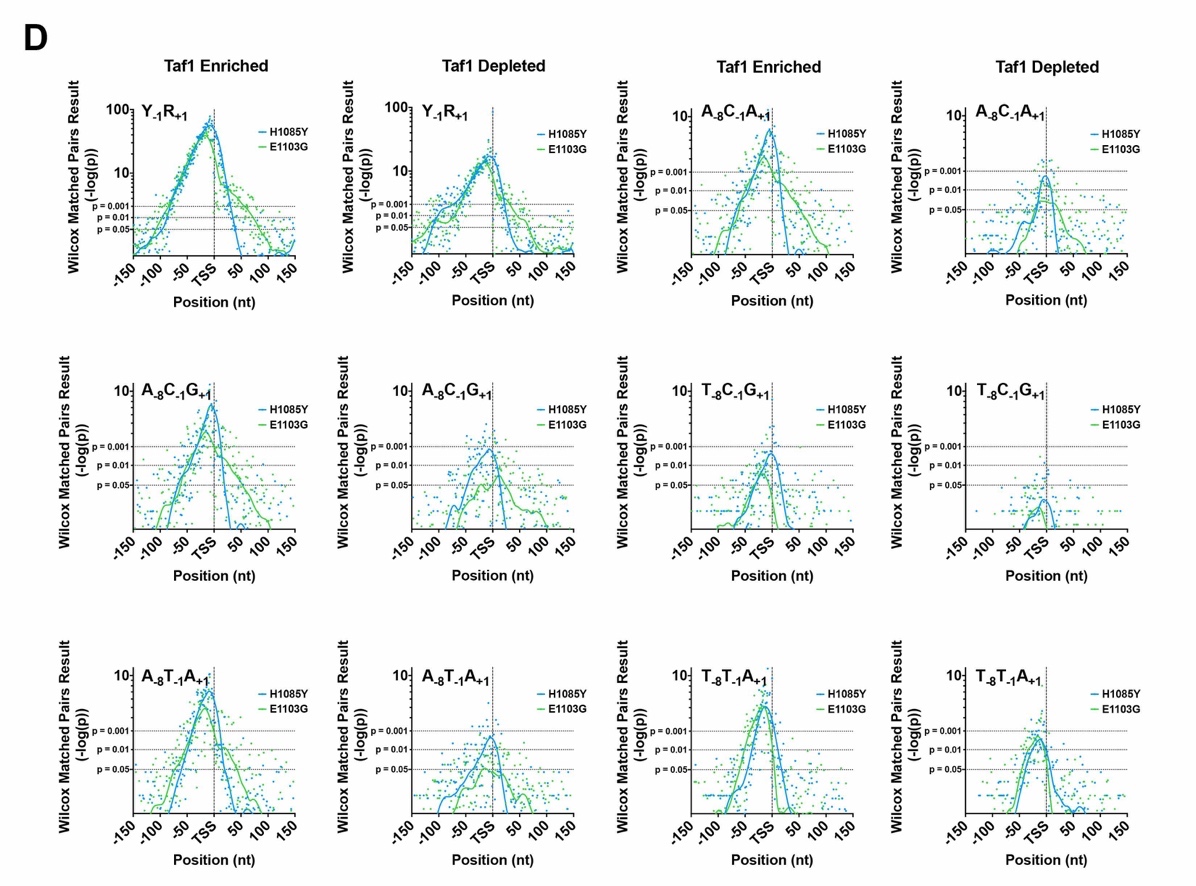


**Fig. S5.** TSS-shifting mutants alter TSS usage and efficiency across TSS motifs throughout promoter regions. **a.** Example median TSS usage (left) for A_-8_C_-1_A_+1_ motifs for WT, *rpb1* E1103G, and *rpb1* H1085Y strains, or TSS efficiency for A_-8_C_-1_A_+1_ motifs for the same strains (right) across Taf1 Enriched promoters. Median usage/efficiency determined from the subset or promoters that have an A_-8_C_-1_A_+1_ motif at the designated promoter position (see schematic in **Fig. 4c**). **b.** Same as (**a**) but for Taf1 Depleted promoters. **c.** Statistical analysis of distributions of TSS Usage between WT and *rpb1* E1103G, or WT and *rpb1* H1085Y strains for specified motif at each promoter position. The Wilcoxon Matched-Pairs Signed Rank test was used to compare distributions of WT usage of a particular motif for a particular promoter position with the distributions of usage for those motifs/positions in Pol II mutants. Therefore, there is a p-value determined for each promoter position for each motif. The -log(p) of each p-value is displayed on the *y*-axis of a plot for a number of example motifs, separated into Taf1 Enriched and Depleted promoters. The lines are LOWESS smooths of individual points. Note that some positions will have no motifs across promoters at a particular position and significance will in part be determined by how many instances of motif there are at each position across promoters. Almost all motifs shown exhibit clusters of positions with significant differences between mutants and WT. **d.** Analysis as in (**c**) but for TSS efficiencies.


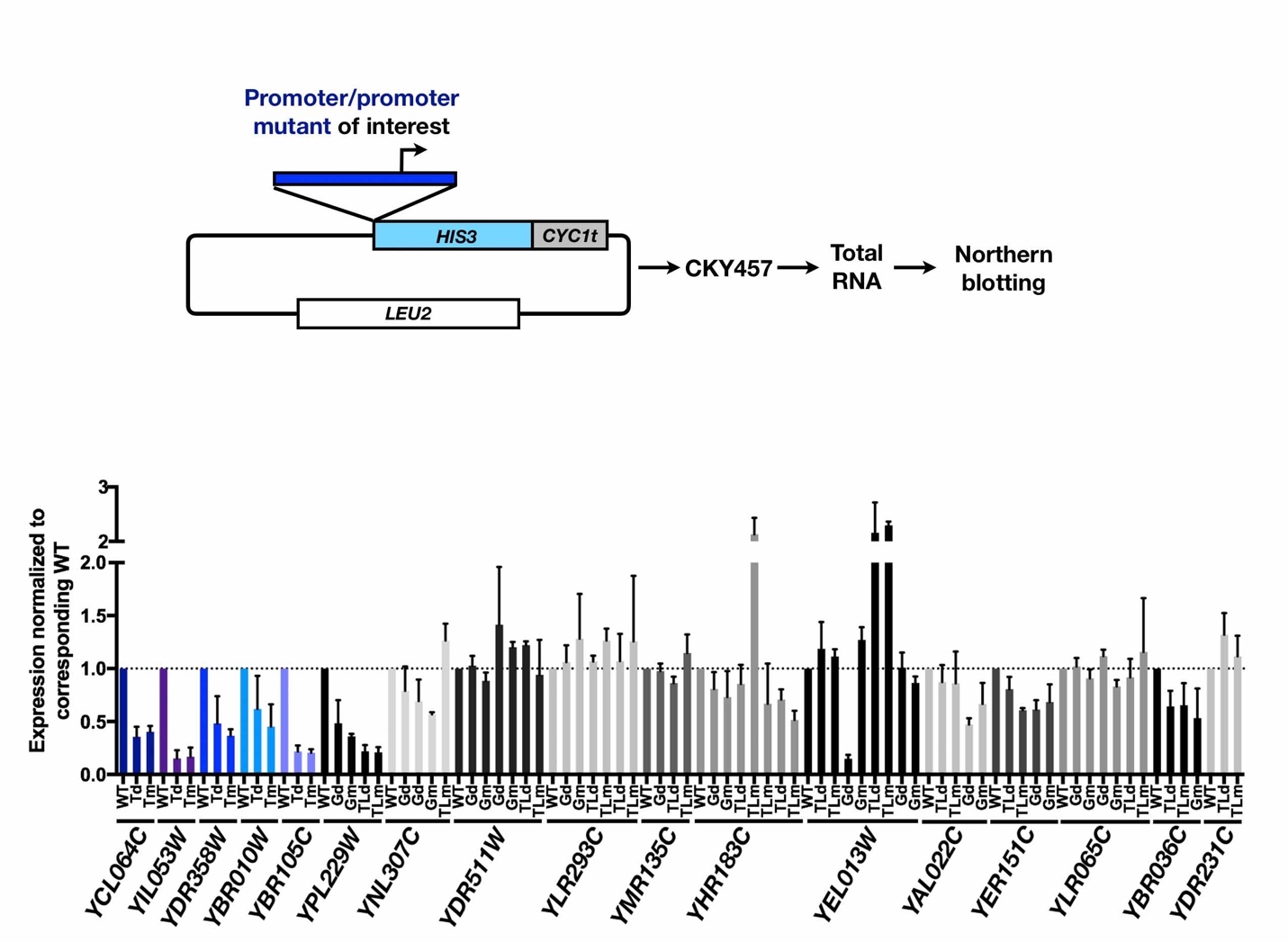


**Fig. S6.** Effects on expression level of putative core promoter element mutations. (Top) Schematic of reporter plasmids fusing promoters of interest (up to ATG) to a *HIS3* ORF/*CYC1* terminator reporter. (Bottom) Quantification of Northern blotting for control WT or promoters mutated (“Tm”) or deleted (“Td”) for consensus TATA elements (promoters shaded in blue), mutated or deleted for GAE (“Gm” or “Gd”, respectively) or mutated or deleted for TATA-like elements identified by Rhee and Pugh or our own analyses (“TLm”, “TLd”, respectively). Bars are mean +/- standard deviation of the mean (n=≥3).

**
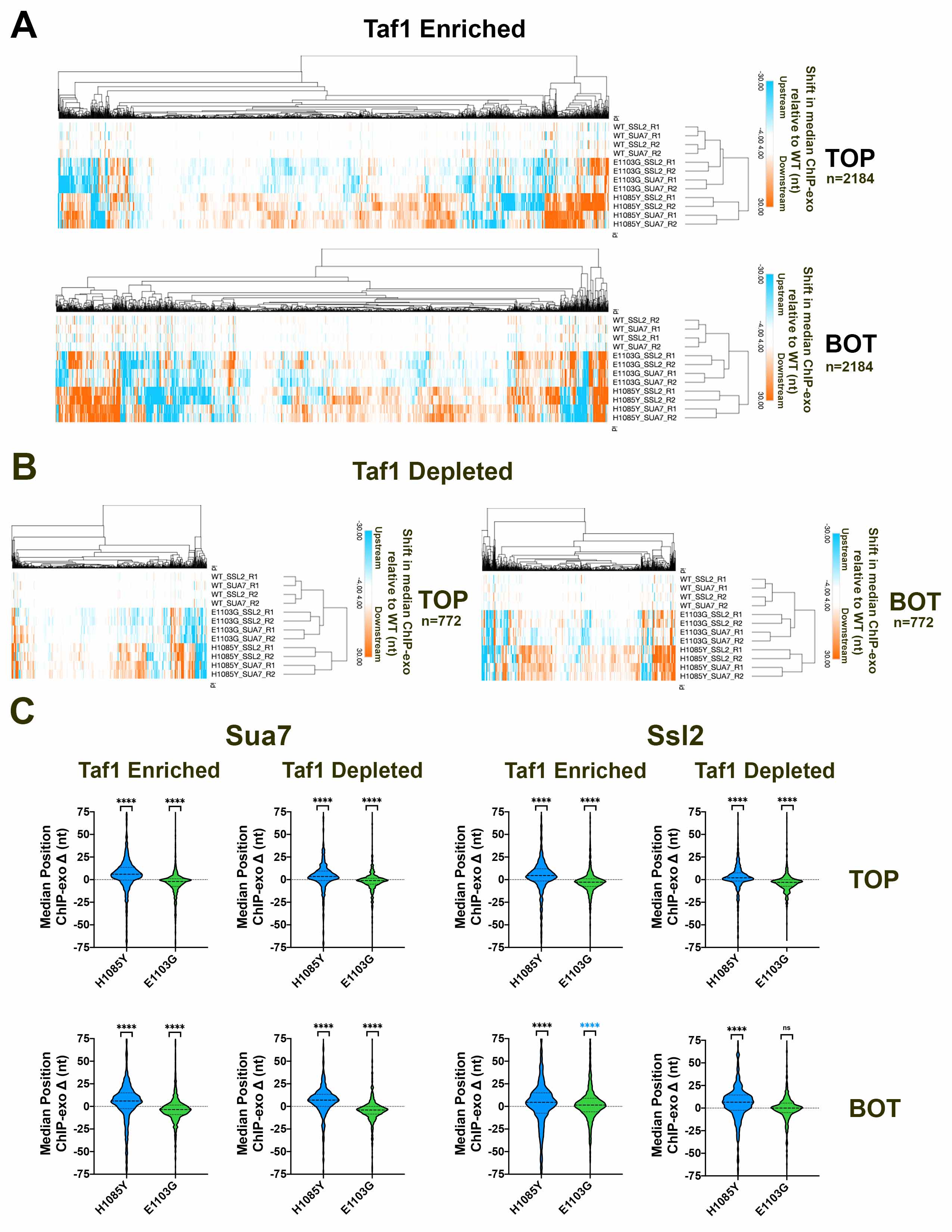
**

**Fig. S7.** ChIP-exo analysis if Sua7 and Ssl2 in *rpb1* catalytic mutants. **a.** Heat map of median ChIP-exo value shifts relative to WT for individual ChIP-exo replicates for top (TOP) or bottom (BOT) DNA strand signal for Taf1 Enriched promoters representing the top ~50% of overall ChIP-exo signal as determined by WT signal for each factor. ChIP-exo signal medians were determined for each promoter window on both strands for two biological replicates for each strain. Median positions were determined for individual replicates and the WT average position was subtracted. A positive value (orange) indicates that a mutant has a downstream shift in ChIP-exo signal while a negative value (cyan) indicates an upstream shift. **b.** Same as in (**a**) but for Taf1 Depleted promoters. **c.** Statistical analysis of average shift in median ChIP-exo signal on top (TOP) or bottom (BOT) DNA strands for Sua7 or Ssl2 at Taf1 Enriched promoters (left) or Taf1 Depleted promoters in WT or *rpb1* mutants. Promoter-mapped ChIP-exo tags compared for two biological replicates for WT, *rpb1* E1103G, and *rpb1* H1085Y in Ssl2-TAP and Sua7-TAP strains. Median of averaged shifts in *rpb1* H1085Y or *rpb1* E1103G compared to zero (no shift) by Wilcoxon Signed Rank Test (**** indicates p<0.0001). Note that Ssl2 for Taf1 Enriched BOT in E1103G shifts downstream 1 nt (marked with blue asterisks) and this is the opposite shift than for all other significant effects.


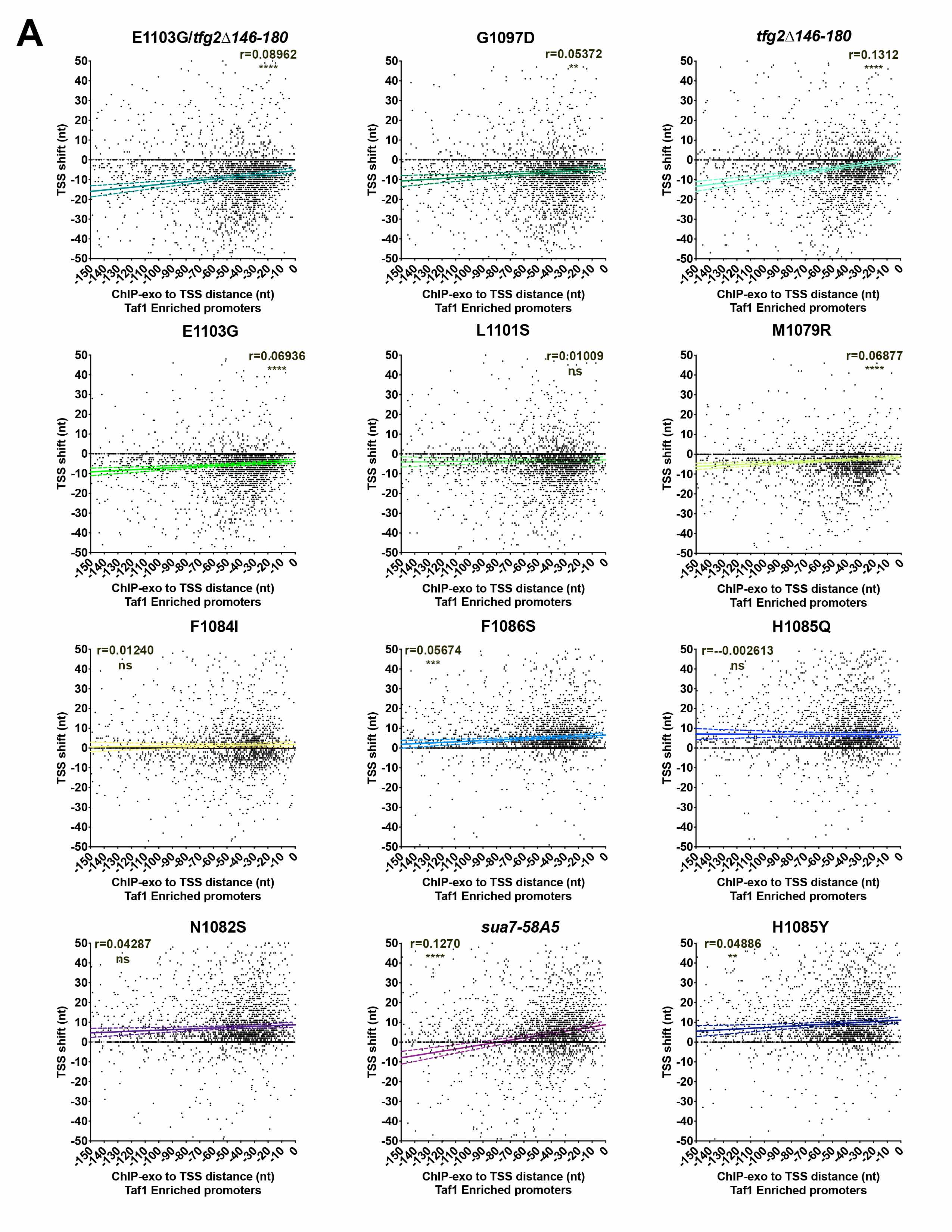

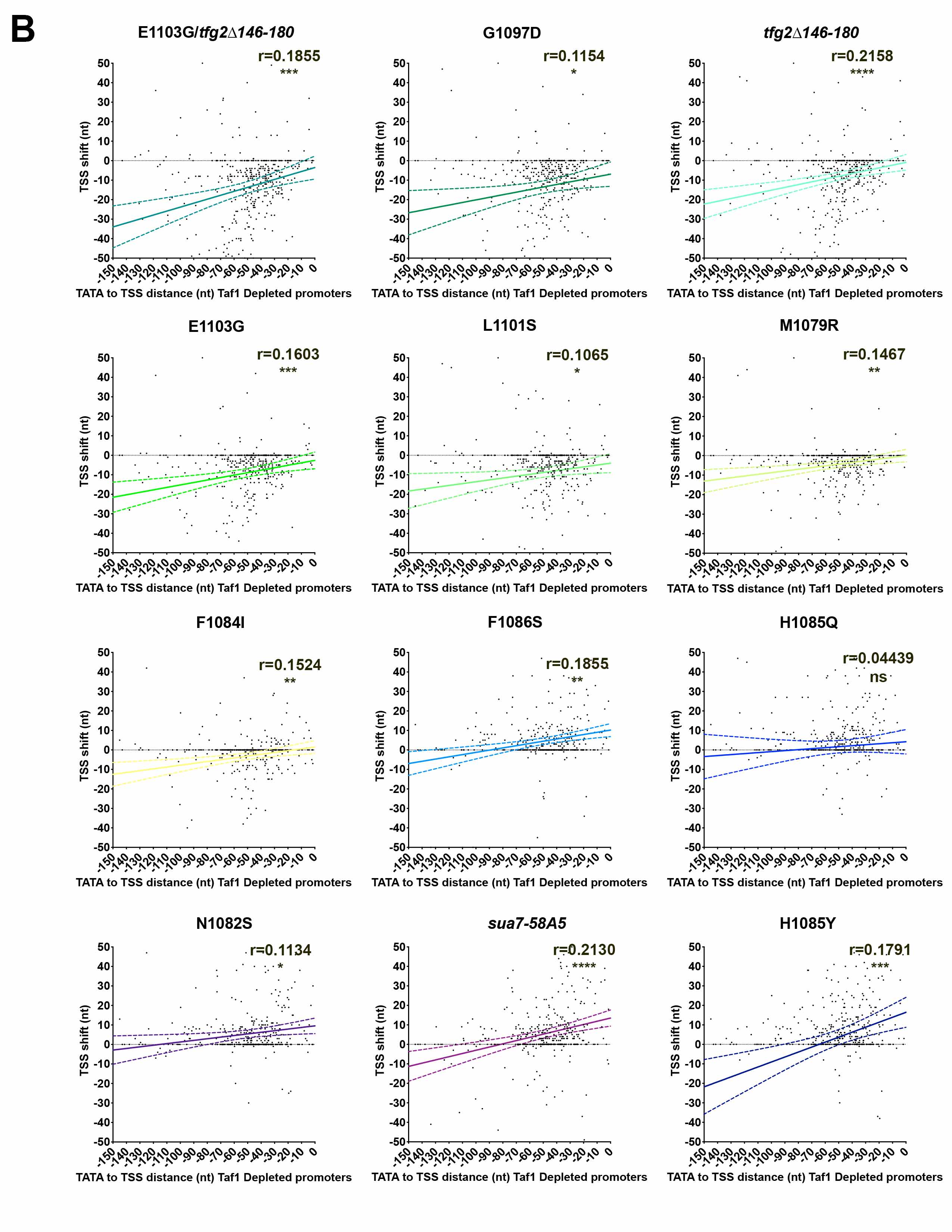


**Fig. S8.** Correlation of TSS shift with ChIP-exo or core promoter element-TSS distance. **a.** Median TSS shifts (*y-*axis) for promoters ≥ 100 reads expression in WT for denoted TSS mutants for Taf1 Enriched promoters plotted versus ChIP-exo-TSS distance (*x*-axis). Lines are linear regression with 95% confidence interval for the linear fit. Pearson r correlations are shown for each plot with asterisks indicating P value (two-tailed, (0.0332 (*), 0.0021 (**), 0.0002 (***), <0.0001 (****)). **b.** Median TSS shifts (*y-*axis) for promoters ≥ 100 reads expression in WT for denoted TSS mutants for Taf1 Depleted promoters with consensus TATA elements plotted versus consensus TATA-TSS distance (*x*-axis). Lines are linear regression with 95% confidence interval for the linear fit. Pearson r correlations are shown for each plot with asterisks indicating P value (two-tailed, (0.0332 (*), 0.0021 (**), 0.0002 (***), <0.0001 (****)).


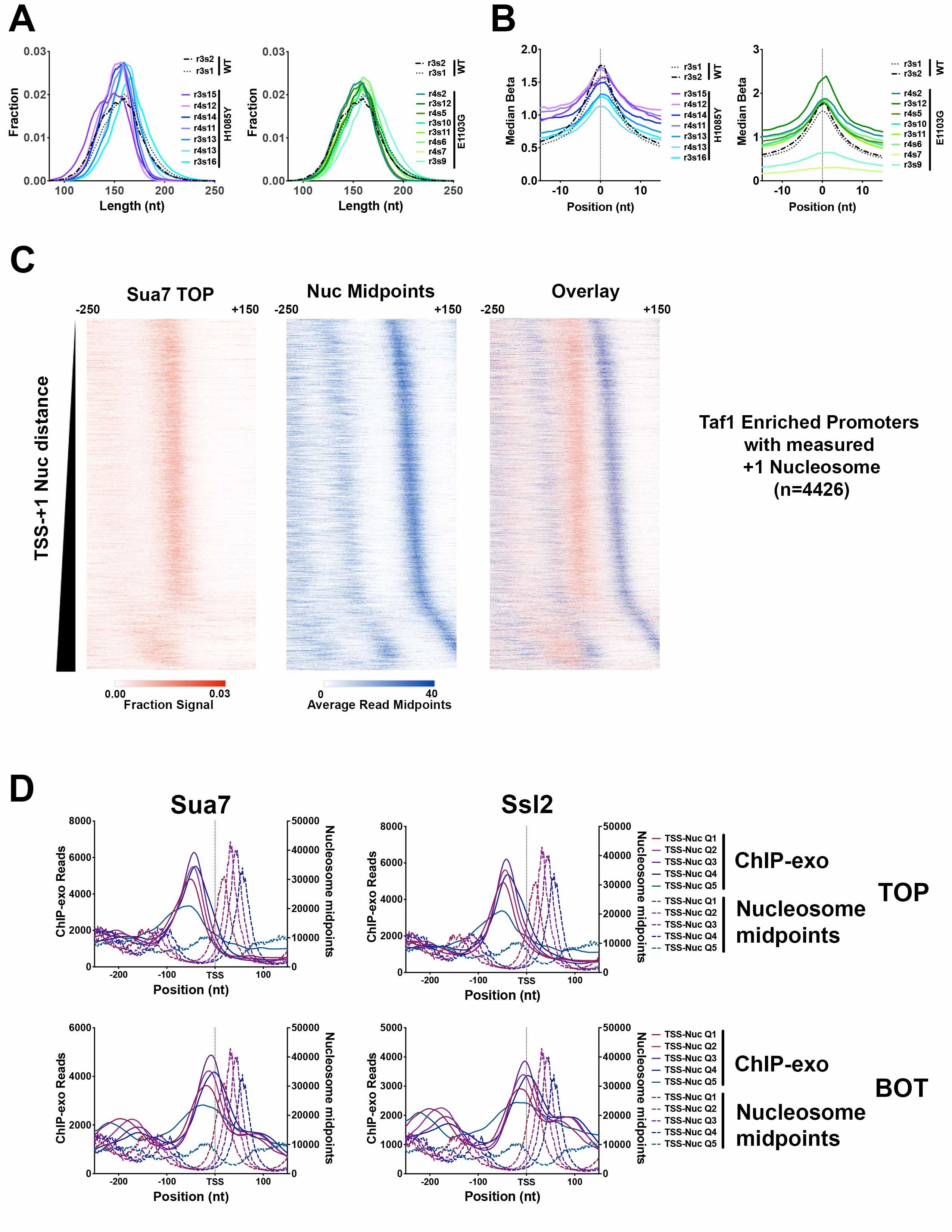


**Fig. S9.** MNase-seq analyses of nucleosome positions in WT, *rpb1* H1085Y, and *rpb1* E1103G mutants. **a.** Paired-end sequencing fragment length distributions in WT and H1085Y MNase-seq libraries (left) and in WT (as left, shown for reference) and E1103G MNase-seq libraries (right). Libraries arranged within groups from most digested (top) to least digested (bottom). **b.** Probability of nucleosome positioning (“Beta”) values determined by method of Zhou *et al* for MNase-seq libraries arranged as in **a**. **c.** Heat maps of Sua7 TOP strand ChIP-exo signal (right), nucleosome +1 midpoints (middle) or overlay of the two (left) indicating correlation of PIC component localization and nucleosome positioning for Taf1 Enriched promoters. **d.** Nucleosome midpoints as determined by MNase-seq (dashed lines) and GTF ChIP-exo signals for Taf1 Enriched promoters (solid line LOWESS smooth of scatter plots) were aggregated by promoter quintiles determined by TSS-+1 nucleosome midpoint position. Nucleosome midpoints are from WT strain and the same data are shown as reference for each ChIP-exo plot. First to fifth quintiles are promoters with the closest +1 nucleosome to furthest, respectively. Fifth quintile promoters likely have a weak +1 nucleosome and thus the determined +1 nucleosome is in some cases like the +2. ChIP-exo aggregate data shows intermediate correlation with +1 nucleosome-TSS distance.

**
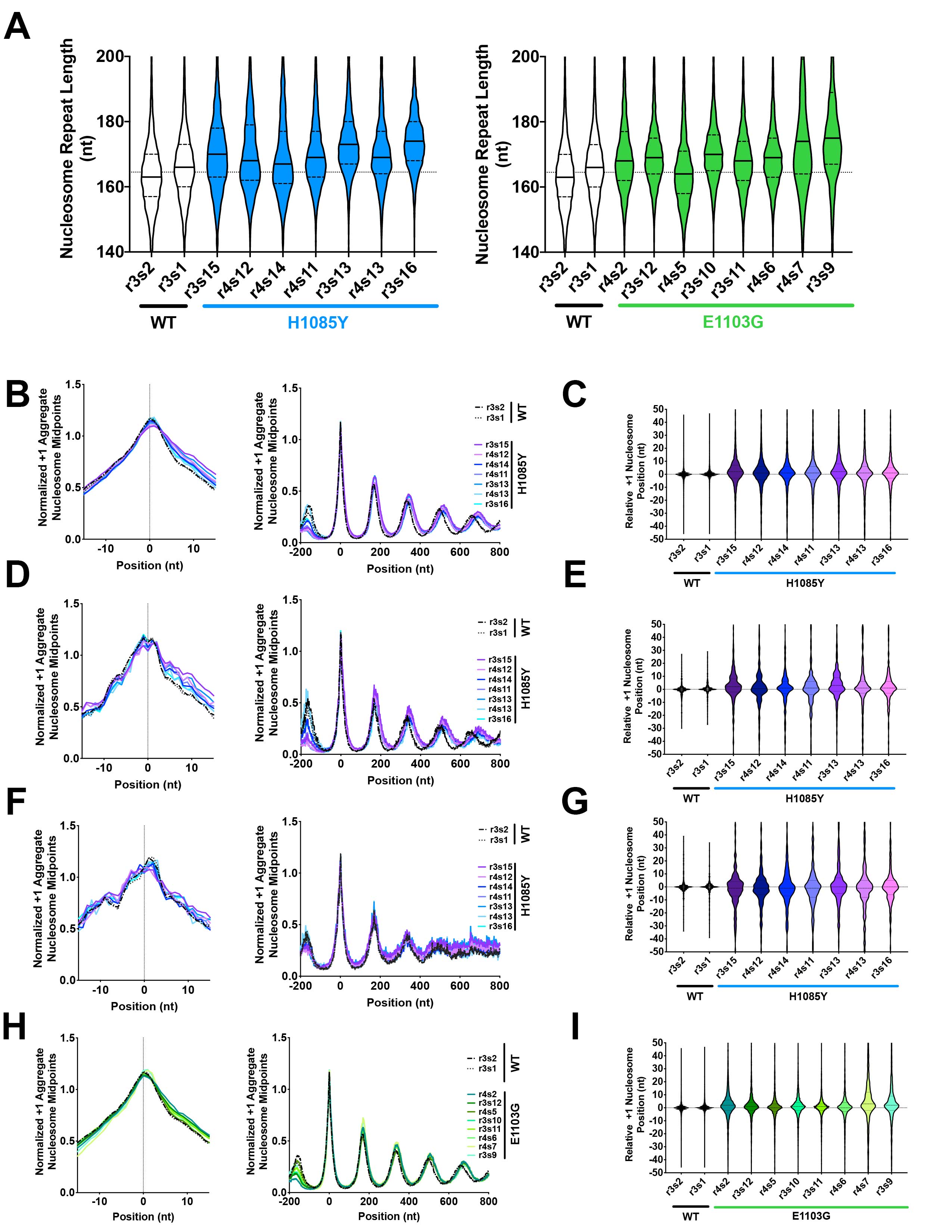
**

**Fig. S10.** Relationship of promoter chromatin architecture to PIC position and effects of TSS-usage affecting mutants on nucleosome positioning. **a. (Left)** WT MNase-seq replicates (n=2) compared to *rpb1* H1085Y MNase-seq replicates (n=7) for nucleosome repeat length as determined by autocorrelation analysis (see Methods). **(Right)** Same as left but for *rpb1* E1103G vs WT (WT samples same on left). **b.** Nucleosome positioning in WT and *rpb1* H1085Y for Taf1 Enriched promoters aligned by +1 nucleosome in WT (left), over genes (-200 to +800 from +1 nucleosome position, right). **c.** Determined +1 nucleosome position for WT and *rpb1* H1085Y Taf1 Enriched promoters for individual MNase-seq libraries relative to position determined by averaging the four WT libraries. Box plots are Tukey plots (see Methods). **d**, **e.** Nucleosome positioning analyses as in (**b, c**) for top expression decile Taf1 Enriched promoters for WT and *rpb1* H1085Y. **f**, **g.** Nucleosome positioning analyses as in (**b**, **c**) for bottom expression decile Taf1-enriched promoters for WT and *rpb1* H1085Y. **h**, **i.** Nucleosome positioning analyses as in (**b**, **c**) for Taf1 Enriched promoters for *rpb1* E1103G. WT data from (**b**, **c**) shown as reference.
