# Supplementary material for: Universal promoter scanning by Pol II during transcription initiation in Saccharomyces cerevisiae
Source: Genome Biol. 2020 Jun 2;21:132. doi: 10.1186/s13059-020-02040-0 (PMC7265651; doi:10.1186/s13059-020-02040-0)
Supplement: Supplementary file 5 — Additional file 5. Review history. [file 13059_2020_2040_MOESM5_ESM.docx]

Review History

**First round of review**

**Reviewer 1**

**Are you able to assess all statistics in the manuscript, including the appropriateness of statistical tests used?** Yes, and I have assessed the statistics in my report.

**Comments to author:**

Review of promoter scanning during.. By Qui et al.

This paper is focused on understanding the underlying logic of TSS distributions within promoters. The fundamental experiment is to sequence capped 5' ends of RNAs in RNAPII and GTF mutants with altered RNAPII elongation rates and/or processivity,   and then assessing whether the TSS distribution changes, and then relating that to models of how RNAPII select TSSs. This is an important question that has not been answered in the field even though the concept of broad TSS distributions as a very common feature in mammalian genomes was raised over 10 years ago. The authors do this in bakers yeast, which may be considered an odd choice as a model given that it does not have the same TATA-TSS location as most eukaryotes. The authors convincingly show that the different RNAPII mutants (especially fast and slow subtypes of RNAPII) lead to 'shifts' in TSS distributions that are consistent with a model where RNAPII scans the promoter. They then extend this to also investigate whether mutants have different nucleosome positions and spacing, and/or shifts of GTFs.

General thoughts

This is a really important subject and the general experimental idea is very sound, from the generation of TSS-seq for mutants to the extension to exo-ChIp.
The largest weakness of the manuscript is that it feels unfinished, and as a result is very challenging to read and understand - the text is often hard to read and imprecise, key features are missing such as informative figure legends, units in figures, colors are not explained, deposited data cannot be found,  and it is unclear whether there are more than duplicates made of sequencing experiments. Moreover some of the computational analysis is very non-standard, and there is a general lack of statistical analyses.  There are also part of the analyses that are not convincing. We feel that the paper should be rewritten and the data at least partially reanalyzed, with the help of dedicated bioinformatics groups.


Major

General issues
Images often lack color legends, units on axes and generally have non-informative figure legend texts. This means that the reader has to guess what the different elements and colors are, this also makes it difficult to assess the effect size in some of these plots. This is not acceptable. For example, just in figure 1, panels B,C, D, E have these issues.

Related: The color scales of some heat maps may give a biased impression, becasue the authors chose a narrow range of colors, which makes results with very small effect size look the same as results with bigger effect size. For example, in Figure 1D, 2A, 3B, 4B, 4C.  
Bioinformatics and statistical analyses seem to have been made by many different people using a vast diversity of tools, many of which are non-standard or obsolete. Some parts of methods are hard to understand.

Top-bottom strand. The choice of referring to +/- strand as top bottom was very confusing. This is the first paper  we have seen that does that. I would propose using +/-, forward reverse, or similar so that it is clear that strand is referred to - especially in images.

Lack of examples or conceptual overview figures which might help readers to understand when the analysis is complicated. For example, in  Figure 3A, it would be easier to understand the analysis if the authors include a cartoon to explain what does primary/secondary… TSS mean.

The text and analysis up to figure 4 are much better and connected than the remaining figures, where some are not convincing and others are not very connected to the rest in terms of narrative.

Specific issues
Page 5: It is very unclear how many replicates that are made. The accession ID for NCBI projects is not working, and we could not find the information elsewhere, more that it is duplicates or more. Generally speaking, to do any serious statistical analysis with RNA-sequencing technology, triplicates or more are required - it is not possible to assess variance of single data points otherwise.  Given how cheap it is to do sequencing and that the authors use yeast, there are no real excuses to only do duplicates.

Page 5: The authors show correlation plots to demonstrate high reproducibility in Fig 1B and S1-2. These plots are not terribly impressive (and lack color legends) as such, and lack correlation scores. More generally, it would be more informative to make a PCA plot of all replicates and samples, possibly on tag cluster level. This would show what libraries that are similar and if replicates cluster where they should.  This is more helpful than the correlation heat map in 1C and S2. The S1-S2 figures are hard to understand and evaluate  since the library names are not explained, nor are the X and Y axes units in S1.  

Figure 1D: Why is the plot centered on  (predicted) TATA boxes rather than the TSSs (which are experimental evidence)? Given that the goal of the figure seems to be to show that the experimentally defined TSSs fall downstream of TATA boxes, the heat map representation may be too complex, especially given that the TAF-1 enriched cases are many more than the other classes. Use simple density plots showing TSS-TATA distances instead? Incidentally, what happens if several TATA-like patterns are predicted?

Figure 1E: the left heat map is a key figure in the manuscript and quite persuasive.  However, it should be complemented with proper statistics. In how many promoters does the median shift have such strength that it is significant (see e.g. https://doi.org/10.1186/gb-2006-7-12-r118 , that does a similar analysis on human data) , and what is the variance between replicate libraries in terms of shifts?  This will likely require triplicates of each library.

Figure 2B: the effect size difference (boxplot medians) is not large. Is this significant?(Also, colors of boxplots are not explained)

Figure 2D. Why is there a 'hole' in the x axis between 2 and 4? Legend says 'Elongation rates are from XXX'. Confusing.

Page 6-7:
The authors claim "Upstream TSS shifting mutants...show a decreased preference for A-8Y-1R+1 motifs..."(line 253-256). Would it be more direct to compare if there is any change of preferences in those TSSs with upstream/downstream shifting showing in figure 1E?

Similarly, the idea of using triplet DNA sequences is nice. But, instead of just clustering triplet usage across mutants, it would be much better to (also?) make a transition matrix for those promoters that show (significant) shifts. That would show the actual changes rather than average preferences. Similarly, why not make logos for respective mutant binding preferences?

Figure 3D. Given that the A in the A8C-1A+1 motif is not very strong (and, actually, the pY Pu is not a 100% occurrence), it may be more appropriate to do this analysis with a weight matrix instead (like, one of the logos from A) . It could even be done with weight matrices for respective mutant, which would likely show the same pattern  but more convincingly show how the mutant would affect the shift in sequence space.

Page 7-8. The analysis of efficiency of motifs and TSSs in interesting  but could be explained better in main text. For figure 4A, are these shifts statistically significant? It would also be helpful for non-yeast readers to say that the different mutants do (slow or fast RNAPII etc). This is true for nearly all figures. For figures 4B-C, colors are never explained, and there is no unit. The mutant names are very hard to read also.

Supplementary Figure 6. Either an y=x trend line should be shown in the plot or the x, y axes should have the same scale. From this plot,  it looks like there are some systematic biases in the ChIP-exo experiment between replicates. For example,  the Ssl2 H1085Y, replicate2 has overall much higher reads/promoter window than replicate1.

Figure 5C. Some variance estimates are needed. Also, are these shifts significant? Also, the figure text can easily be misunderstood so that the reader believes that the lines are different promoter classes - they are not.  

Figure 5D. The shifts are very modest and at times not believable - are these really significant?

Figure 6. None of these correlation analyses are convincing. Please remove this analysis and its text. There are also no statistical analyses presented .

Figure 7A: These plots are almost unreadable because of the many line types and double axes.  Also, if the point is to see the relation between GTFs and MNase peaks, it would be better to make a correlation as a function of distance plot, because in the current plot, we do not know if the peaks co-occur since it is just the sum or average.

Figure 7B-H. The shifts are not convincing: the effect size is small and there are no statistical analyses made. Similar to above, the proper analysis for proving increased spacing is an autocorrelation by distance analysis (so, the distance between consecutive peaks) - that way one looks at co-occurrence rather than aggregates over all promoters.  
The boxplots are also very similar in terms of medians - except for possibly in panel I, they are not convincing, and as above, no statistics has been done.

Figure 8 and associated text. These analysis are not very convincing and are not very connected to the rest of the paper. To start with, the same methodology is not used - CAGE data exist for all of these species, so why not use that? Moreover, initiation spread is a function not only of biology but also number of reads, so sequencing depth per promoter should be taken into account (eg by random sampling). Moreover, selecting subsets of data (eg non-SP in S pombe) makes the data even less comparable.  If the authors want to make these points, they have to do it with comparable data sets and the same bioinformatics methodology for mapping, cluster calling etc. The methods section for the part is also confusing.

Minor issues

At least two citations are not double checked ('XXX' in one instance and "cite Schwalb …" in another)

The first 6 cited papers are all reviews from Kadonaga et al. These are great, but it may be more balanced to include reviews by other authors as well.

Page 6. The 64 'motifs' are not motifs - a motif is a pattern which typically has some flexibility so we often model it with a consensus or a matrix representation. They are k-mers or triplets  if anything.

Page 5: "We note that a fraction of promoter windows has TSSs arising from positions suggesting that the responsible PICs for those TSSs assemble at additional positions, either upstream or downstream." This is not understandable. TSSs arising from positions?

Page 5: "Promoters were two-dimensionally clustered". Very unclear. Does this just mean that the authors cluster per promoter (rows) and libraries/mutants(columns) for each heat map?

Page 8-9. The TATA-nonTATA section does, when you read it, have nothing to do with the overall narrative. It becomes clearer that this is needed for the next section, but it is confusing at first sight. Perhaps merge these two sections or improve the narrative?

**Reviewer 2**

**Are you able to assess all statistics in the manuscript, including the appropriateness of statistical tests used?** Yes, and I have assessed the statistics in my report.

**Comments to author:**

In this manuscript, Qui et al. performed a detailed study of the transcription start site (TSS) usage in S. cerevisiae. The authors analyze genome-wide TSS usage in multiple mutants with and increase/decrease catalytic activity for the RNApol II (fast/slow). This works expands their previous work and show that in general fast polymerases (GOF) tend to use upstream TSS independent of the promoter class. Their data support that a change of RNA pol II activity (independent of motif), together with an asymmetric distribution across the genome and the directionality of the transcription process is sufficient to explain the differential use of TSS motifs across strains with different catalytic activity. To complement that work, the authors perform a detailed study of Sua7, Ssl2 and nucleosomes in the polymerase mutants for GOF and LOF. Finally, the authors try to expand some of their conclusion to other eukaryotes.

I found this work rigorous, and I think it would be of general interest for the transcription community. In addition, it provides a very rich dataset that will be of wide interest for researchers working in transcriptional regulation in yeast. The only concern I have is the expansion to other eukaryotes (Fig8), that I feel is weaker in comparison to the rest of the manuscript. I do not think that last part is essential, and I think the detailed work in S. cerevisiae should be sufficient for this publication. To improve the manuscript, I have the following recommendations.

Major:
1.     Line 508. The effect described in Fig 8A could be explained by sampling bias (low express genes have less reads and thus the detected promoters will be in general less spread). Once the coverage is sufficient, the distribution is expected to stabilize, but not before. To reach the conclusion that low express reads are less spread, the authors should subsample random sequencing reads from high expressed genes down to the numbers observed in the low expressed genes. That improved dataset will be better suited to answer the proposed question.

2.     Fig 8B. Continuing the previous point. Were the reads per promoter in S. pombe at a similar depth level than in cerevisiae. Or could that be explained just by different coverage as before?

3.     Fig 8C-D.  Was the data controlled by the relative AT richness of the different promoters across species? All those figures seem to simply show the trivial relationship that longer pieces of DNA (spread) contains more nt inside (and thus more YR). I am not sure if I am missing something… Also, as spread is associated with detection of TSS, and TSS is very frequently YR that could also explain Fig 8E. I found more interesting Fig S10.

Minor:
4.     Fig 1E. I imagine the data in grey are genes without sufficient reads. That should be described in the figure legend or removed from the heatmap.

5.     Fig 2C. The legend is not sufficient to explain what is depicted in this figure. For example, E and D are not described.

6.     Line 253. The authors describe that upstream shifting mutants show a decrease preference for A-8Y-1R+1 (Fig 3B). They show that there is a general effect of the TSS usage independently of the motif analyzed (Fig4) and suggest that the distribution is likely cause by the more frequent use of upstream TSS and uneven distribution of motifs across the promoter region. However, there is an additional feature that could contribute to that effect. It has been previously described in yeast that TSS separated by exactly 8 nt are overrepresented (eg. Fig S2 in PMID: 23615609). If this observation holds in the authors dataset, this will predict that the A+1 position of an upstream TSS might in some cases contribute to the A-8 signal observed genome wide. And thus, explain also why an increase usage of upstream TSS (first in the pair) tends to decrease the presence of A-8. This is a minor point, but it might contribute to strengthen the authors model.

7.     Fig 4A and 4C are very complex to understand. The definition of median % usage should be improved. Either in the figure legend or by adding some model in the supplementary material.

8.     Fig 4. It is very difficult to visualize. The authors use a scale of blue and greens, that is very useful to refer to the different mutants. However, this color scheme, together with the shades of all data points, makes impossible to visualize the data.  I would advise to move this figure to supplementary and provide a simplified version for the main text without the data points.

9.     Fig 4D-E. To my understanding this figure shows that fast polymerases tend to use upstream TSS and slow downstream ones (as Fig1). Basically, in the x-axes 0 means upstream and -150 downstream. A similar picture would be obtained if a random position (eg. distance from nucleosome +1, or -1) was used. I would recommend removing these 2 panels, or moving them to supplementary.

10.     Fig7A. It is very difficult to visualize. I would move this to supplementary and show in the main text one plot without the data points.

11.     A section including the norther blot and cloning strategy described in figure S5 should be added to the methods.

12.     In general figure font sizes and resolution should be improved.

13.     Line 1463. There is a citation missing marked by (XXX).

**Authors Response**

**Point-by-point responses to the reviewers’ comments:**

*We thank the reviewers for their careful, thoughtful review and their investment in time evaluating our work. Their comments have allowed us to improve the manuscript in accessibility, readability, and scientific rigor. We have endeavored to address all comments below (our responses are in italics). To summarize our revisions, we have added statistical analyses across the manuscript, and streamlined the manuscript by removing figures deemed distracting. We note that in general all our previously presented results are statistically significant. The big picture of our experiments is that Pol II and initiation factor mutants have effects across almost all promoters. These are apparent in aggregate analyses and for individual promoters. Mutants are of two major classes, upstream and downstream shifting. Consistent effects are observed for many mutants of each class, reinforcing conclusions for any individual mutant. Pol II mutants also alter global chromatin structure in terms of nucleosome spacing as apparent from metaplots of chromatin and is obvious from visual analysis of heat maps of MNase-protected fragment midpoints. Autocorrelation analysis of the data confirms an increase in nucleosome spacing for Pol II mutants tested.*

**Reviewer #1**: Review of promoter scanning during.. By Qui et al.

This paper is focused on understanding the underlying logic of TSS distributions within promoters. The fundamental experiment is to sequence capped 5' ends of RNAs in RNAPII and GTF mutants with altered RNAPII elongation rates and/or processivity, and then assessing whether the TSS distribution changes, and then relating that to models of how RNAPII select TSSs. This is an important question that has not been answered in the field even though the concept of broad TSS distributions as a very common feature in mammalian genomes was raised over 10 years ago. The authors do this in bakers yeast, which may be considered an odd choice as a model given that it does not have the same TATA-TSS location as most eukaryotes. The authors convincingly show that the different RNAPII mutants (especially fast and slow subtypes of RNAPII) lead to 'shifts' in TSS distributions that are consistent with a model where RNAPII scans the promoter. They then extend this to also investigate whether mutants have different nucleosome positions and spacing, and/or shifts of GTFs.

General thoughts

This is a really important subject and the general experimental idea is very sound, from the generation of TSS-seq for mutants to the extension to exo-ChIp.

The largest weakness of the manuscript is that it feels unfinished, and as a result is very challenging to read and understand - the text is often hard to read and imprecise, key features are missing such as informative figure legends, units in figures, colors are not explained, deposited data cannot be found,  and it is unclear whether there are more than duplicates made of sequencing experiments. Moreover some of the computational analysis is very non-standard, and there is a general lack of statistical analyses. There are also part of the analyses that are not convincing. We feel that the paper should be rewritten and the data at least partially reanalyzed, with the help of dedicated bioinformatics groups.

Major

General issues

Images often lack color legends, units on axes and generally have non-informative figure legend texts. This means that the reader has to guess what the different elements and colors are, this also makes it difficult to assess the effect size in some of these plots. This is not acceptable. For example, just in figure 1, panels B,C, D, E have these issues.

***We have redone figure legends and have paid attention especially to legends and scales, with justifications of choices for visual analysis and scaling.***

Related: The color scales of some heat maps may give a biased impression, because the authors chose a narrow range of colors, which makes results with very small effect size look the same as results with bigger effect size. For example, in Figure 1D, 2A, 3B, 4B, 4C.

***Thanks to the reviewer for making this point. For the visual presentation of the data, it was not our goal to obscure effect sizes by color, but to make trends that are reproducible across a large number of mutants of both classes visually obvious. For example, Figure 1D was adjusted to show where the reads are across windows. We have made it clear in the legend that these are normalized (by row) TSS distributions and that even with the color bar indicating usage down to a fraction of a percent, the TSSs are relatively highly localized across promoters. The heat maps have more data points that the resolution of the figure can show, therefore there will also be some averaging in the figures.***

***Figure 2A shows difference maps. The effects are small at many individual sites and because effects are distributed unevenly relative to position of the major TSS, and also because at this resolution figure will have some averaging, a universal effect could appear diminished based on the scale chosen. The reduced scale we have chosen makes the key result obvious that there is a polar effect in TSS usage at essentially all promoters. We think that heat maps coupled with Figure 2B make this point and we do not mean to imply magnitude of effects are greater than they are. We would like to note that shifting distributions of TSSs at essentially all promoters, even if modest is a global effect consistently observed in essentially all mutants in a class-specific manner (Supplemental Figure 2), where fast mutants consistently shift the TSS upstream while slow mutants consistently shifts the TSS downstream in almost all promoters. We feel our manuscript goes far in explaining a plausible mechanism. We must also point initiation is changing globally and it is unclear to what extent greater defects would even be tolerated in vivo. Finally, the double mutant between* rpb1 *E1103G and tfg2∆146-180 exacerbates defects across the genome quite strongly. Statistical analyses have been added to all box/violin/distribution plots. We also add a number of additional analyses in main and supplemental figures indicating the reproducibility of the results and the robustness across multiple mutants of each class.***

***For Figure 3B, heatmap is colored so that differences are visually apparent, not to obscure differences. For example, in 3B the point is that mutants cluster into two main groups and show directional effects for the same TSS groups.***

Bioinformatics and statistical analyses seem to have been made by many different people using a vast diversity of tools, many of which are non-standard or obsolete. Some parts of methods are hard to understand.

***We have streamlined the manuscript and attempted to revise methods in a way where the manuscript is consistent and more accessible. We have discussed our analytical approaches with a number of informaticists.***

Top-bottom strand. The choice of referring to +/- strand as top bottom was very confusing. This is the first paper we have seen that does that. I would propose using +/-, forward reverse, or similar so that it is clear that strand is referred to - especially in images.

***This is a comment where we are in disagreement with the reviewer, but do our best here to explain why the usage needs to be precise as we have it. We have added language and a new schematic (Figure 5C) to make this more clear. Promoters have an intrinsic directional orientation independent of genome strand. Because we take + and – strand to indicate the + and – strands of the genome, we differentiate the two strands of a directional sequence (promoter) as top and bottom because these strands are not + and -. A + strand promoter will have the + strand as the top strand and the – strand as the bottom strand, and vice versa for – strand promoters. This is because Top and Bottom are defined relative to the orientation of the promoter and have the same orientation relative to upstream and downstream. In contrast, forward and reverse strands have their own 5’ to 3’ orientations. We think it is essential to keep promoter strand orientation separate from other terminologies for distinguishing strands.***

Lack of examples or conceptual overview figures which might help readers to understand when the analysis is complicated. For example, in  Figure 3A, it would be easier to understand the analysis if the authors include a cartoon to explain what does primary/secondary… TSS mean.

***Great idea. We have added these types of schematics to multiple figures.***

The text and analysis up to figure 4 are much better and connected than the remaining figures, where some are not convincing and others are not very connected to the rest in terms of narrative.

***We address this point in comments below, but we have streamlined the latter portions of the manuscript. We have endeavored to tighten the narrative as well.***

Specific issues

Page 5: It is very unclear how many replicates that are made. The accession ID for NCBI projects is not working, and we could not find the information elsewhere, more that it is duplicates or more. Generally speaking, to do any serious statistical analysis with RNA-sequencing technology, triplicates or more are required - it is not possible to assess variance of single data points otherwise.  Given how cheap it is to do sequencing and that the authors use yeast, there are no real excuses to only do duplicates.

***We apologize for not making the NCBI bioproject public until publication, and we have created a reviewer link (***[***https://dataview.ncbi.nlm.nih.gov/object/PRJNA522619?reviewer=3s0sm85our9gl7cfe1pefqcue***](https://dataview.ncbi.nlm.nih.gov/object/PRJNA522619?reviewer=3s0sm85our9gl7cfe1pefqcue)***) for our revision. We previously included a table summarizing all the datasets used in this study, but have now made this more clear. We have 3 or more replicates for WT and for at least 2-3 representative mutants from each class (4 biological replicates for WT, 4 biological replicates for slow mutant F1086S, 3 biological replicates for slow mutants H1085Y, 4 biological replicates for fast mutants M1079R, E1103G and G1097D). Given the correlations between multiple polymerase mutants in each class and correlation of individual datasets for each mutant, and the clear clustering of mutant types by a number of analyses (e.g. PCA and hierarchical clustering), we are loath to add any additional repeats. If these were simple RNA-seq experiments, we agree that we would have done more and could immediately add a number of repeats. However, in this specific case, we are constrained for a few reasons. First, the primary cost for library preparation in this approach was that the particular TSS-seq methodology employed used a large amount of rRNA-depleted total RNA, made with a cost-prohibitive method (RiboZero) that Illumina has now discontinued for yeast. Newer methodologies would require an alteration to the protocol, which we are using for other experiments. Furthermore, our sequencing for this particular project was SOLiD-based. The entirety of the project would need to be repeated with a new protocol and different sequencing technology for any additional repeats to be added, with potential batch effects we can foresee. It would not be economical from the point of view that the results will not qualitatively change. To address these issues in a different way, we now add supplemental figures showing the correlation of TSS metrics performed on individual libraries along with PCA plots for these metrics. We note that individual libraries correlate well with each other, and PCA differentiates classes of mutant and clusters repeats based on mutant type (fast or slow). PCA does have the ability to identify batch effects across our two major sequencing runs, but our experimental design had multiple replicates partitioned and well-balanced across batches for the majority of samples across two different mutant classes.***

Page 5: The authors show correlation plots to demonstrate high reproducibility in Fig 1B and S1-2. These plots are not terribly impressive (and lack color legends) as such, and lack correlation scores.

***We note that the correlation plots are at the level of individual bases in the genome and not cluster or feature (promoter). If we plotted versus feature the correlations would go up. As such almost all Pearson r for individual positions in genome (with applied threshold of ≥3 reads per position in each of compared libraries) are already mostly ≥0.7. We have added Pearson r and color legend for the heat scatter plots for all plots.***

More generally, it would be more informative to make a PCA plot of all replicates and samples, possibly on tag cluster level. This would show what libraries that are similar and if replicates cluster where they should.  This is more helpful than the correlation heat map in 1C and S2. The S1-S2 figures are hard to understand and evaluate since the library names are not explained, nor are the X and Y axes units in S1.

***We have added color scales and units to all graphs. We have added PCA plots for a number of metrics. We have explained library names in figure legends, have noted them in Additional File 2 where there is information cross-referencing individual replicates with their library designation.***

Figure 1D: Why is the plot centered on (predicted) TATA boxes rather than the TSSs (which are experimental evidence)? Given that the goal of the figure seems to be to show that the experimentally defined TSSs fall downstream of TATA boxes, the heat map representation may be too complex, especially given that the TAF-1 enriched cases are many more than the other classes. Use simple density plots showing TSS-TATA distances instead? Incidentally, what happens if several TATA-like patterns are predicted?

***The explanation for the analysis was that our starting point for promoter definition was promoters defined by Rhee and Pugh based on ChIP-exo of factors (Rhee and Pugh 2012). We wish to illustrate where actual transcription is found relative to putative core promoter elements designated by Rhee and Pugh. Here TATA or TATA-like are designated based on their occurrence with a ChIP-exo signature. We note later in the manuscript that TATA-like prevalence is pretty high across promoters. Subsequent to this initial analysis, which was shown to illustrate the logic of our approach, we realigned these Rhee/Pugh promoter windows based on the experimentally observed TSSs (Figure 2A), and these are the windows used for the rest of the analyses.***

Figure 1E: the left heat map is a key figure in the manuscript and quite persuasive.  However, it should be complemented with proper statistics. In how many promoters does the median shift have such strength that it is significant (see e.g. https://genomebiology.biomedcentral.com/articles/10.1186/gb-2006-7-12-r118, that does a similar analysis on human data), and what is the variance between replicate libraries in terms of shifts?  This will likely require triplicates of each library.

***We have performed the statistical analysis recommended for all mutants (shown in Supplemental Figure 3). Based on our prior results with a single promoter (ADH1, Jin et al), where we observed extremely reproducible and quantitative effects of a large number of Pol II mutants and initiation factors, we weren’t surprised that the reproducible TSS-seq also differentiated between mutant classes and WT. The result is that essentially all mutants show a large number of individual promoters affected and a large bias in direction of effect. We have analyzed significance in two ways for individual promoters, by the method suggested (Kruskal Wallis with Dunn’s Correction to ask if median TSS position is significantly different between a mutant promoter or WT promoter for each promoter) and additionally by Mann Whitney U test. Note that each of these tests analyzes changes to median TSS positions in biological replicates, and median itself is already a conservative metric depending on how much of a promoter’s TSS usage is present in the median TSS. Furthermore, there is a huge bias in direction of promoters significantly shifting in individual mutants (upstream vs downstream).***

Figure 2B: the effect size difference (boxplot medians) is not large. Is this significant? (Also, colors of boxplots are not explained)

***We apologize for not explaining the color scheme. The mutants are colored by their relative strengths/in vitro elongation rates from blue to green (slowest to fastest) and have now noted this color scheme in the legend. Non-parametric Wilcoxon Signed Rank test indicates all mutant distribution medians differ from that of hypothetical value of zero (p < 0.0001) except for F1084I in Taf1-depleted promoters (ns). Additionally, all slow mutants are statistically distinct from all fast mutants if examined using Kruskal-Wallis with Dunn’s correction for multiple testing (adjusted P values <0.0001, not shown).***

Figure 2D. Why is there a 'hole' in the x axis between 2 and 4? Legend says 'Elongation rates are from XXX'. Confusing.

***The reference to the elongation rates for these mutants has been added (we had duplicated figure legends and missed the duplicate legend). We have added a note to the legend that the gap merely indicates we have used a different scale for the x axis between 1 and 2 so that mutants in that region can be spread out for ease of analyses.*  *We have added the linear regression line for the correlation and the metric for goodness of fit (R* *squared).***

Page 6-7:

The authors claim "Upstream TSS shifting mutants...show a decreased preference for A-8Y-1R+1 motifs..."(line 253-256). Would it be more direct to compare if there is any change of preferences in those TSSs with upstream/downstream shifting showing in figure 1E?

Similarly, the idea of using triplet DNA sequences is nice. But, instead of just clustering triplet usage across mutants, it would be much better to (also?) make a transition matrix for those promoters that show (significant) shifts. That would show the actual changes rather than average preferences. Similarly, why not make logos for respective mutant binding preferences?

***This is somewhat shown in Supplemental Figure 4, changes in motif usage across individual promoters separated into class, ranked by expression in wild type for two mutants. Because individual promoters also have their own unique sequence composition, for example more highly expressed promoters can be T-rich upstream, there is a greater gain of TTA usage for E1103G at these promoters but that is potentially because of the increased frequency of TTA motif for those promoters in upstream positions. Because almost all promoters show a shift either up or downstream, and promoters have their own unique sequence context (available TSSs to switch to within the scanning window) it may be difficult to deconvolute further. We favor the analysis in the revised Figure 4, showing that when controlling for promoter position essentially all efficiently used motifs show alterations in efficiency consistent with the scanning model. Separate experiments we are doing now in the follow up to this project by analyzing massive promoter libraries of defined compositions will answer these questions definitively in the future. Logos for mutant TSS preferences will look qualitatively similar because ACA remains the preferred motif.***

Figure 3D. Given that the A in the A8C-1A+1 motif is not very strong (and, actually, the pY Pu is not a 100% occurrence), it may be more appropriate to do this analysis with a weight matrix instead (like, one of the logos from A). It could even be done with weight matrices for respective mutant, which would likely show the same pattern but more convincingly show how the mutant would affect the shift in sequence space.

***PyPu is not 100% but it is actually quite high (~90%, previous Supplemental Figure 10A). Our analysis of TSSs based on efficiency, which in a scanning process is likely the best way to compare TSSs rather than usage, which is dominated by priority effects, indicates that -8AYR TSSs are more efficient on average when accounting for promoter position (distance from promoter core). If a YR is present, -8A is a major driver of efficiency, and ACA TSSs are the most efficient on a positional basis out of all motifs. As noted above, given that promoters have unequal nucleotide and therefore TSS motif distributions, alterations to TSS sequence preference can’t be determined just from genome usage, which is why we turned to the efficiency analysis shown in 4E. 4D shows that usage is altered when attempting to normalize to sequence and 4E shows that efficiency “envelopes” change regardless of individual motifs analyzed and that ACA/ACG/ATA TSSs are each on average (median), regardless of promoter position. We have added statistical analyses of these changes to usage and efficiency distributions (Supplemental Figure 5) comparing distributions of usages/efficiencies at each promoter positions between two representative mutants and WT. Though the curves described by the median usage and efficiency appear close to WT, these differences are due to highly significant shifts in the underlying populations of usages and efficiencies.***

Page 7-8. The analysis of efficiency of motifs and TSSs is interesting but could be explained better in main text. For figure 4A, are these shifts statistically significant? It would also be helpful for non-yeast readers to say that the different mutants do (slow or fast RNAPII etc). This is true for nearly all figures. For figures 4B-C, colors are never explained, and there is no unit. The mutant names are very hard to read also.

***We thank the reviewer here and throughout the document for their attention to detail. It is greatly appreciated. The units are “percent efficiency” or “percent normalized usage” and have been added to the heat maps. For 4D colors are positive (gain) or negative (loss) in normalized usage. We have added schematics to explain these analyses better and have updated the text. We observe directional shifts in aggregate usage for all mutants and examined TSS motifs, regardless if overall (bulk) usage of a motif goes up or down. As noted above, these changes have been analyzed for statistical significance and in fact are highly significant.***

Supplementary Figure 6. Either an y=x trend line should be shown in the plot or the x, y axes should have the same scale. From this plot, it looks like there are some systematic biases in the ChIP-exo experiment between replicates. For example, the Ssl2 H1085Y, replicate2 has overall much higher reads/promoter window than replicate1.

***We have repeated ChIP-exo using the latest generation ChIP-exo v5.0 (two repeats per sample) versus the previous (v1). We have removed the old data. The new repeats show essentially the same results but the data are of much higher quality and signal-to-noise ratio. We have revised all ChIP-exo figures showing the new data and have presented it somewhat differently.***

Figure 5C. Some variance estimates are needed. Also, are these shifts significant? Also, the figure text can easily be misunderstood so that the reader believes that the lines are different promoter classes - they are not.

***The purpose of 5C was to show that our simple metric of determining median position of ChIP-exo signal for different factors on each strand gave distributions of values consistent with expectations for PIC factor binding. Signal upstream of TSS, Sua7 on average upstream of Ssl2, For ChIP-exo, we only have two biological replicates for v5.0 ChIP-exo. However, this methodology gives highly consistent data between replicates (see new figures).***

Figure 5D. The shifts are very modest and at times not believable - are these really significant?

***The shifts are modest but reproducible and significant (see new analyses). Our philosophy is that we would present the data regardless of the result. We show shifts in aggregate (metaplot) and on the distribution of individual promoter measurements (violin plots). The extent of shifts are consistent with the nature of TSS shifts, i.e. shift in TSS usage is a shift in probability of use for TSSs that are observed (for the most part) at some level in WT. This means a shift in distribution of signal ChIP-exo within regions where signal is already observed. The effects are greater for rpb1 H1085Y, showing effects on Ssl2 and Sua7, with Ssl2 on bottom strand shifting also significant in rpb1 E1103G (see new figures).***

Figure 6. None of these correlation analyses are convincing. Please remove this analysis and its text. There are also no statistical analyses presented.

***The correlations for the most part are significant and this analysis has been retained but only in part. The correlations are not expected to be strong for these analyses given that there are many other reasons for changes in expression (analysis removed), and that each promoter has individual sequence context that will determine strength of shift (analysis retained). The trend in shift vs core promoter position is consistent across all mutants with trend in same direction even for mutants that have opposite shifts,* i.e. *the slopes are the same sign. The slopes are those predicted from a scanning mechanism where being close to core promoter should limit upstream shifting and being far from promoter should limit downstream shifting. We have removed the expression analysis for the most part because that is not a major focus of the work. The distance analysis we retain because the results are robust. Furthermore, they supports a prediction of scanning model – promoters with TSSs evolved at marginal (near or far) distances from core promoter elements could be differentially sensitive to perturbation to scanning. We have moved these analyses to Supplemental Figure 7, noting the Pearson r , statistical significance, and individually showing linear regression fits and 95% confidence intervals.***

Figure 7A: These plots are almost unreadable because of the many line types and double axes.  Also, if the point is to see the relation between GTFs and MNase peaks, it would be better to make a correlation as a function of distance plot, because in the current plot, we do not know if the peaks co-occur since it is just the sum or average.

***The peaks co-occur given that a large fraction of promoters have significant ChIP-exo signal and an even larger fraction of promoters have positioned nucleosomes. More importantly, this analysis simple recapitulates PIC-nucleosome relationship demonstrated in Rhee and Pugh (2012) (shown there in heat map and metaplot). We have cleaned up the plots as requested while also showing an example heat map.***

Figure 7B-H. The shifts are not convincing: the effect size is small and there are no statistical analyses made. Similar to above, the proper analysis for proving increased spacing is an autocorrelation by distance analysis (so, the distance between consecutive peaks) - that way one looks at co-occurrence rather than aggregates over all promoters.

The boxplots are also very similar in terms of medians - except for possibly in panel I, they are not convincing, and as above, no statistics has been done.

***The shifts are modest but statistically significant. We have added the statistical tests indicating significance p<0.0001 (expected even for small effects due to the large number of measurements). We would report these data even if there were no effect as the result is the result. In this case the result is there is an apparent subtle but reproducible shift in +1 position, but a much more obvious alteration to nucleosome repeat length (in both tested mutants). We have added autocorrelation analysis to determine nucleosome repeat lengths in WT and mutants. These lengths are statistically significantly different from WT. Median +1 nucleosome positions are non-zero and positive (downstream shift) for all H1085Y datasets, for example.***

Figure 8 and associated text. These analysis are not very convincing and are not very connected to the rest of the paper. To start with, the same methodology is not used - CAGE data exist for all of these species, so why not use that? Moreover, initiation spread is a function not only of biology but also number of reads, so sequencing depth per promoter should be taken into account (eg by random sampling). Moreover, selecting subsets of data (eg non-SP in S pombe) makes the data even less comparable.  If the authors want to make these points, they have to do it with comparable data sets and the same bioinformatics methodology for mapping, cluster calling etc. The methods section for the part is also confusing.

***We have removed these analyses but clarify our motivation here. In some species, older CAGE data as analyzed were suspect at the individual TSS level due to additional G residues added in the CAGE protocol (dependent on RT enzyme used and temperature at which it is employed). More recent CAGE data do not have these issues as predicted additional Gs are removed due to informatics analyses. In order to renanalyze data from scratch, and given Reviewer 2’s feelings about these analyses, we have removed 8B-8E from the manuscript in current form. This being said, analysis of published TSS distributions is a reasonable approach without needing to generate new data. Remapping with the same pipeline is reasonable though there is no great expectation that general distributions in terms of spread will be highly sensitive to mapping pipeline. We also note that non-SP are merely promoters that are not a single TSS (these essentially are the only meaningful pombe promoters to examine as they are the class that are represented by multiple TSSs).***

Minor issues

At least two citations are not double checked ('XXX' in one instance and "cite Schwalb …" in another)

***Thank you for catching these. We have proofed and fixed.***

The first 6 cited papers are all reviews from Kadonaga et al. These are great, but it may be more balanced to include reviews by other authors as well.

***Jim Kadonaga has dominated core promoter reviews recently with regards to Inr usage, but we have added some additional reviewers there, including Haberle and Stark and some others.***

Page 6. The 64 'motifs' are not motifs - a motif is a pattern which typically has some flexibility so we often model it with a consensus or a matrix representation. They are k-mers or triplets if anything.

***Because our TSS groups are (-8)NNNNNN(-1)(+1), they seem like they can be considered motifs because of the intervening Ns. We feel that motif is reasonable here for that reason.***

Page 5: "We note that a fraction of promoter windows has TSSs arising from positions suggesting that the responsible PICs for those TSSs assemble at additional positions, either upstream or downstream." This is not understandable. TSSs arising from positions?

***We mean that TSSs identified at locations positioned such that the presumed PIC location would not physically be possible to generate the observed TSSs. This result was due to likely experimental noise in the PIC determination pipeline from Rhee and Pugh. We have rewritten this section.***

Page 5: "Promoters were two-dimensionally clustered". Very unclear. Does this just mean that the authors cluster per promoter (rows) and libraries/mutants(columns) for each heat map?

***Yes, we meant that the promoters and libraries (rows and columns) were both hierarchically clustered. We have revised the text.***

Page 8-9. The TATA-nonTATA section does, when you read it, have nothing to do with the overall narrative. It becomes clearer that this is needed for the next section, but it is confusing at first sight. Perhaps merge these two sections or improve the narrative?

***We have tried to clarify our reasoning here and why this was important to address. The section has been revised.***

**Reviewer #2:** In this manuscript, Qui et al. performed a detailed study of the transcription start site (TSS) usage in S. cerevisiae. The authors analyze genome-wide TSS usage in multiple mutants with and increase/decrease catalytic activity for the RNApol II (fast/slow). This works expands their previous work and show that in general fast polymerases (GOF) tend to use upstream TSS independent of the promoter class. Their data support that a change of RNA pol II activity (independent of motif), together with an asymmetric distribution across the genome and the directionality of the transcription process is sufficient to explain the differential use of TSS motifs across strains with different catalytic activity. To complement that work, the authors perform a detailed study of Sua7, Ssl2 and nucleosomes in the polymerase mutants for GOF and LOF. Finally, the authors try to expand some of their conclusion to other eukaryotes.

I found this work rigorous, and I think it would be of general interest for the transcription community. In addition, it provides a very rich dataset that will be of wide interest for researchers working in transcriptional regulation in yeast. The only concern I have is the expansion to other eukaryotes (Fig8), that I feel is weaker in comparison to the rest of the manuscript. I do not think that last part is essential, and I think the detailed work in S. cerevisiae should be sufficient for this publication. To improve the manuscript, I have the following recommendations.

Major:

1.    	Line 508. The effect described in Fig 8A could be explained by sampling bias (low express genes have less reads and thus the detected promoters will be in general less spread). Once the coverage is sufficient, the distribution is expected to stabilize, but not before. To reach the conclusion that low express reads are less spread, the authors should subsample random sequencing reads from high expressed genes down to the numbers observed in the low expressed genes. That improved dataset will be better suited to answer the proposed question.

***The reviewer is right that sampling can affect these measures, however, the more highly expressed promoters have the narrower spread- we have now made more clear that expression decile 1 represents the most highly expressed promoters. Given this is the fraction of promoters where we have a large number of reads, the results are not likely to be affected (and given that the lowly expressed promoters are more highly spread, sampling will undoubtedly return this same result). We have both labeled the figure more clearly to indicate expression decile 1 are the highest expressed promoters and 10 are the lowest.***

2.    	Fig 8B. Continuing the previous point. Were the reads per promoter in S. pombe at a similar depth level than in cerevisiae. Or could that be explained just by different coverage as before?

***Because of both Reviewer 1 and 2 concerns that these analyses don’t really add to the paper in current form, we have removed most of Figure 8 from this manuscript while we analyze data from scratch along the lines of Reviewer 1s comments for inclusion elsewhere. We respond here to Reviewer 2 comments here for the sake of illuminating the issues and explaining our logic. We used a read cutoff to mitigate many issues of sequencing depth. Given that TSS-seq and CAGE are relatively low noise for bona-fide TSS clusters, once above this noise, distributions should be relative stable to sampling. Pombe coverage was not different from cerevisiae, and others have also noted that pombe promoters are “tighter”/”narrower” than yeast, and by extension also narrower than other species.***

3.    	Fig 8C-D.  Was the data controlled by the relative AT richness of the different promoters across species? All those figures seem to simply show the trivial relationship that longer pieces of DNA (spread) contains more nt inside (and thus more YR). I am not sure if I am missing something… Also, as spread is associated with detection of TSS, and TSS is very frequently YR that could also explain Fig 8E. I found more interesting Fig S10.

***These analyses have been removed to streamline the manuscript. We respond here to illuminate for the reviewer what the analyses indicated. Figure 8E controlled for AT richness indirectly, as it examine the  use of non-CG YR. The critical result here is actually the slope of YR usage relative to the increase in spread. For example, zebrafish maternal and zygotic promoters have different slopes here even though these TSS distributions can be adjacent or overlapping for promoters that are both maternally and zygotically expressed. This slope is a way of expressing TSS usage density relative to potential TSSs. The idea being that scanning will “see” all TSSs in a region, whereas other mechanisms (independent specification of TSSs from distinct PICs) will not “see” all TSSs and therefore might be expected to have a lower density of usage. Human promoters have a shallower slope as well (indicating lower density of TSS usage for diffuse promoters) but this is likely due in part to CpG richness (a non-preferred YR element). Our analyses in C/D get at an intriguing result from Haberle et al from a different angle. Haberle et al noticed in their supplemental figures that maternal TSS clusters had wider intra-cluster TSS-TSS distances than zygotic. We showed in our analyses that these differences were not due to YR density differences and or putative TSS availability. This was an interesting result because it suggested that zebrafish maternal promoters are “spikier” (similar TSS spreads but lower density TSSs within the spreads) but not for lack of YR elements.***

Minor:

4.    	Fig 1E. I imagine the data in grey are genes without sufficient reads. That should be described in the figure legend or removed from the heatmap.

***Corrected. Thanks to the reviewer for noticing this.***

5.    	Fig 2C. The legend is not sufficient to explain what is depicted in this figure. For example, E and D are not described.

***Corrected. Thanks for catching this. E and D are Taf1-enriched and depleted as in B. We have added to the legend and written out the words entirely in the figure.***

6.    	Line 253. The authors describe that upstream shifting mutants show a decrease preference for A-8Y-1R+1 (Fig 3B). They show that there is a general effect of the TSS usage independently of the motif analyzed (Fig4) and suggest that the distribution is likely cause by the more frequent use of upstream TSS and uneven distribution of motifs across the promoter region. However, there is an additional feature that could contribute to that effect. It has been previously described in yeast that TSS separated by exactly 8 nt are overrepresented (eg. Fig S2 in PMID: 23615609). If this observation holds in the authors dataset, this will predict that the A+1 position of an upstream TSS might in some cases contribute to the A-8 signal observed genome wide. And thus, explain also why an increase usage of upstream TSS (first in the pair) tends to decrease the presence of A-8. This is a minor point, but it might contribute to strengthen the authors model.

***Very astute point. Because -8A enhances efficiency of TSS usage of a YR and A itself is the apparent preferred +1 base, the increase in representation of TSSs 8 nt apart is predicted by this arrangement, and absolutely a decrease in usage for the downstream A is predicted. If the -8As do not have their own -8As (as we indicate, they do not on average based on the distribution of A-8N-1A+1 in promoters) then the observed shift in motif usage would be predicted. This is a more specific way of interpreting the effect and we rephrase some language to indicate this – and again we thank the reviewer for this astute and subtle point..***

7.    	Fig 4A and 4C are very complex to understand. The definition of median % usage should be improved. Either in the figure legend or by adding some model in the supplementary material.

***Thank you for this suggestion. We have added a schematic at the beginning of the figure to indicate the basis for our analyses here and have revised the discusstion.***

8.    	Fig 4. It is very difficult to visualize. The authors use a scale of blue and greens, that is very useful to refer to the different mutants. However, this color scheme, together with the shades of all data points, makes impossible to visualize the data.  I would advise to move this figure to supplementary and provide a simplified version for the main text without the data points.

***Given point 9 below, where Figure 4D-E are referenced (which our manuscript did not have), we suspect reviewer is discussing Figure 6 here. We have revised this and removed some portions.***

9.    	Fig 4D-E. To my understanding this figure shows that fast polymerases tend to use upstream TSS and slow downstream ones (as Fig1). Basically, in the x-axes 0 means upstream and -150 downstream. A similar picture would be obtained if a random position (eg. distance from nucleosome +1, or -1) was used. I would recommend removing these 2 panels, or moving them to supplementary.

***We assume reviewer is discussing Figure 6D/E have removed these two panels***

10.  	Fig7A. It is very difficult to visualize. I would move this to supplementary and show in the main text one plot without the data points.

***We have replaced this showing heat map just for nucleosomes but removing datapoints (leaving just the smooth in Supplemental Figures- though the data are new and the figure is easier to interpret).***

11.  	A section including the northern blot and cloning strategy described in figure S5 should be added to the methods.

***Done. Thank you for recommending this.***

12.  	In general figure font sizes and resolution should be improved.

***We have done this in a number of cases. In others, it relates to journal PDF distillation, the original figures are high resolution.***

13.  	Line 1463. There is a citation missing marked by (XXX).

***Thanks for noting this. We have corrected.***

**Second round of review**

**Reviewer 1**

The revised version has improved narrative and made the key points of the study more clear. Allover, we recommend that teh authors try to improve the language further, some sentences are quite long and difficult to understand.

In general, the authors addressed the questions well. A few points recommended for improving the manuscript:


1. To show WT in the violin plots in Fig.6C is redundant, it is actually a violin plot of the distribution of difference between mutant and WT, one only needs the violin plot for the difference; as it is not a real distribution violin plot between WT and mutants. This also means the statistic is off (should test for a null hypothesis of median or mean  change of 0).


2. In general, include the statistical test results (all the p-values) also in the main text, so it would be easier to follow. Also, use actual P values.

**Reviewer 2**

I am satisfied with the author's revision of this manuscript.

**Authors Response**

***We have revised manuscript as requested and dataset links in SRA are now verified as active. We have broken down or rewritten sentences that were long or complex. We have altered Figure 6 as requested by Reviewer 1. We have not added exact p-values as requested by Reviewer 1, as we calculated p-values for most analyses using Graphpad Prism, because Graphpad Prism approximates p-values when datasets are large (as ours are). We think these analyses are reasonable and we feel the Figure legends have attention to statistical detail what will allow readers to interpret what we have done. We added note to methods about nature of statistical analyses. We did calculate exact p-values for statistical analysis of individual promoters and TSS usage by motif by promoter position, but these analyses generated thousands of p-values. As noted, we discovered a processing error in new ChIP-exo analyses. The revised version has corrected Figure 5, where histograms for calculated ChIP-exo medians now look much closer to ChIP-exo version 1 (in our first submission) and there are modest changes to shapes of distribution calculated in meta-promoter aggregate analyses. However, mutant effects are similar or stronger in the corrected analyses. Figure S7A-C, Figure S8-A-B, Figure S9C-D reflect the reprocessed data. In general, changes are modest and conclusions are essentially unaffected.***
